# Discovery of Herbacetin as a Novel SGK1 Inhibitor to Alleviate Myocardial Hypertrophy

**DOI:** 10.1002/advs.202101485

**Published:** 2021-11-10

**Authors:** Shujing Zhang, Yingchao Wang, Min Yu, Ye Shang, Yanxu Chang, Hong Zhao, Yu Kang, Lu Zhao, Lei Xu, Xiaoping Zhao, Dario Difrancesco, Mirko Baruscotti, Yi Wang

**Affiliations:** ^1^ College of Pharmaceutical Sciences Zhejiang University Hangzhou 310058 China; ^2^ State Key Laboratory of Component‐Based Chinese Medicine Tianjin University of Traditional Chinese Medicine Tianjin 301617 China; ^3^ Institute of Bioinformatics and Medical Engineering School of Electrical and Information Engineering Jiangsu University of Technology Changzhou Jiangsu 213001 China; ^4^ School of Basic Medical Sciences Zhejiang Chinese Medical University Hangzhou 310053 China; ^5^ Department of Biosciences University of Milano Milan I‐20133 Italy

**Keywords:** herbacetin, myocardial hypertrophy, *Rhodiola* species, SGK1, traditional Chinese medicine

## Abstract

Cardiac hypertrophy is a pivotal pathophysiological step of various cardiovascular diseases, which eventually leads to heart failure and death. Extracts of *Rhodiola* species (Ext.R), a class of commonly used medicinal herbs in Europe and East Asia, can attenuate cardiac hypertrophy both in vitro and in vivo. Serum/glucocorticoid regulated kinase 1 (SGK1) is identified as a potential target of Ext. R. By mass spectrometry‐based kinase inhibitory assay, herbacetin (HBT) from Ext.R is identified as a novel SGK1 inhibitor with IC_50_ of 752 nmol. Thermal shift assay, KINOMEscan in vitro assay combined with molecular docking proves a direct binding between HBT and SGK1. Site‐specific mutation of Asp177 in SGK1 completely ablates the inhibitory activity of HBT. The presence of —OH groups at the C‐3, C‐8, C‐4’ positions of flavonoids is suggested to be favorable for the inhibition of SGK1 activity. Finally, HBT significantly suppresses cardiomyocyte hypertrophy in vitro and in vivo, reduces reactive oxygen species (ROS) synthesis and calcium accumulation. HBT decreases phosphorylation of SGK1 and regulates its downstream forkhead box protein O1 (FoxO1) signaling pathway. Taken together, the findings suggest that a panel of flavonoids structurally related to HBT may be novel leads for developing new therapeutics against cardiac hypertrophy.

## Graphical abstract

*Rhodiola* species has long history as medicinal and edible plants in Europe and Asia. It is proved that extract of *Rhodiola* species attenuates cardiac hypertrophy via targeting SGK1. Herbacetin is identified as novel SGK1 inhibitor with IC_50_ of 752 nmol, which might be a promising lead compound for treating cardiomyocyte hypertrophy by selectively mediating SGK1‐FoxO1 signaling pathway.

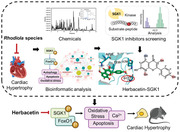

## 1 Introduction

Cardiac hypertrophy is an adaptive process characterized by thickening of the left ventricular wall in response to various intrinsic and extrinsic stimuli. The adaptive hypertrophy helps to reduce hypervolemic stress and maintain cardiac function,^[^ ^1^, ^2^ ^]^ however, this process is limited and will lead to heart failure in the presence of prolonged stress as a consequence of pathological remodeling. Heart failure is a leading cause of mortality and morbidity globally and is the most common cardiovascular reason for hospital admission in populations older than 60 years of age.^[^ ^3^ ^]^ Accompanying pathological cardiac hypertrophy are characterized by increases in cardiomyocyte size, protein synthesis, interstitial fibrosis, and cardiomyocyte death, which are usually preceding pathological events for heart failure and sudden death. The development of cardiac hypertrophy is associated with increased proinflammatory cytokines, hemodynamic overload, myocardial damage, and progressive cardiomyocyte loss, which results in cardiac hypoxia and fibrosis remodeling, and eventually heart failure.^[^ ^4^ ^]^ Many signaling pathways are related to the pathophysiology of cardiac hypertrophy including: the renin‐angiotensin‐aldosterone system (RAAS), the phosphatidylinositol 3‐kinase (PI3K)/Akt pathway, and the mTOR pathway.^[^ ^5^, ^6^ ^]^ Furthermore, studies have revealed essential roles for reactive oxygen species (ROS)^[^ ^7^ ^]^ and the dysregulation of calcium handling^[^ ^8^ ^]^ in cardiac hypertrophic signaling. First‐line treatment of cardiac hypertrophy or heart failure includes angiotensin‐converting enzyme inhibitors or angiotensin II type I receptor blockers (ACE/ARBs), *β*‐blockers, diuretics, and mineralocorticoid receptor antagonists.^[^ ^9^ ^]^ Sodium‐glucose cotransporter 2 (SGLT2) inhibitors and vericiguat (a soluble guanylyl cyclase stimulator) have also been recently proved to improve patients outcomes.^[^ ^10^, ^11^ ^]^ Since complicated mechanisms and pathological processes are involved in the irreversible maladaptive changes during the process of heart failure and remodeling, no simple therapeutic approach is adequate for its prevention or clinical treatment. The discovery of novel therapeutic targets and new drugs for heart failure is still a hot spot in cardiovascular medicine.

Extracts of *Rhodiola* Species (Ext.R) is a remarkable representative of natural products for alleviating cardiovascular illnesses. *Rhodiola* species was classified as a traditional herbal medicinal product by the European Medicines Agency Committee on Herbal Medicinal Products. In a number of European and Asian countries, Ext.R has a long history of bolstering immunity, ameliorating altitude sickness, fighting aging, and reducing burnout in patients with fatigue syndrome. Moreover, clinical studies reported that Ext.R could relieve the symptoms of ischemic heart disease, such as angina pectoris,^[^ ^12^ ^]^ and decrease hypoxia‐induced oxidative stress.^[^ ^13^ ^]^ The major constituents of Ext.R include phenylethanoids, phenylpropenoids, monoterpene glycosides, as well as some flavonoids. However, the effects and the underlying molecular mechanisms of Ext.R on cardiac hypertrophy have not yet been systematically investigated.

Serum/glucocorticoid regulated kinase 1 (SGK1) was thought to play a critical role in maintaining mitochondrial function and cell health. Loss of SGK1 function shortened the lifespan in C. elegans by increasing mitochondrial permeability.^[^ ^14^ ^]^ Inhibition of SGK1 suppressed oncogenic transformation.^[^ ^15^ ^]^ SGK1 was also an important mediator of differentiation of the colorectal cells and inhibited colorectal cancer metastasis.^[^ ^16^ ^]^ SGK1 was first implicated in the development of hypertrophy by Rosenzweig A et al. in 2005.^[^ ^17^ ^]^ The expression of SGK1 in heart tissue is activated in animal models of chronic heart failure and human dilated cardiomyopathy. Cardiac‐specific activation of SGK1 in mice increased mortality rate as well as the incidence of cardiac dysfunction and ventricular arrhythmias, whereas SGK1 inhibition protected mice from fibrosis, heart failure, and sodium channel alterations.^[^ ^18^ ^]^ Although there are commercially available SGK1 inhibitors, i.e., EMD638683 and GSK650394, the discovery of safer and highly specific SGK1 inhibitors with improved pharmacological properties gains great interest in the development of drugs for treating cardiac dysfunction and other associated diseases. However, there is lack of appropriate approach for screening SGK1 inhibitors.

In the present study, we reported Ext.R attenuated pressure‐induced cardiac hypertrophy via inhibiting SGK1 phosphorylation for the first time. A mass spectrometry based assay was developed to evaluate SGK1 activity and screen SGK1 inhibitors from Ext.R. A panel of structurally related flavonoids including herbacetin (HBT) were identified as novel SGK1 inhibitors. Thermal shift assay, KINOMEscan in vitro assay combined with molecular docking were used to reveal the binding domain of HBT and SGK1, and the pharmacological mechanism of HBT against cardiac hypertrophy was further studied.

## 2 Results

### 2.1 Ext.R Protects Mice from TAC‐Induced Cardiac Hypertrophy

**Figure 1:**
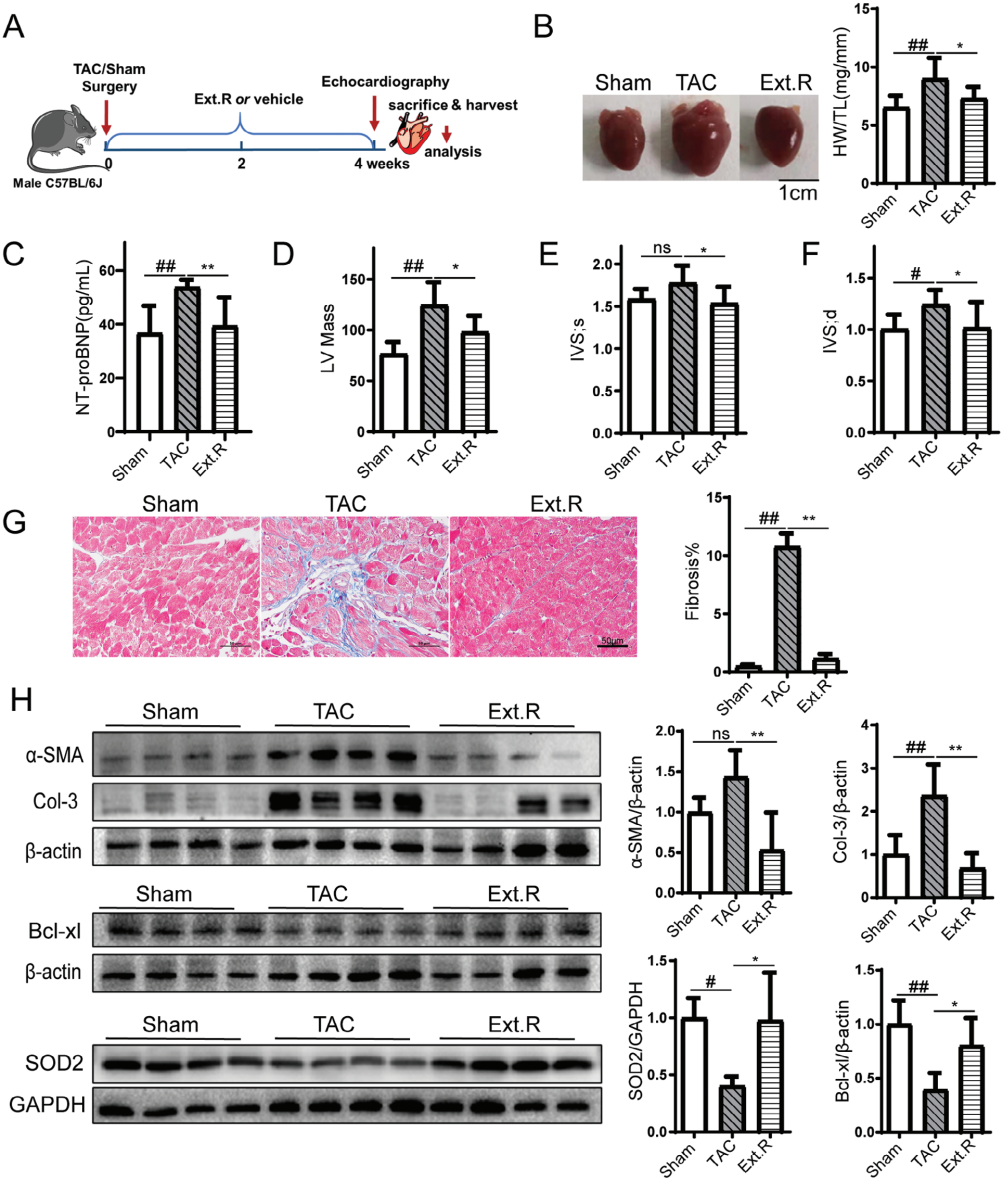
Ext.R blocks cardiac hypertrophic responses in vivo. A) Schedule of animal treatments. Mice were subjected to TAC for 4 weeks in the presence or absence of Ext.R. The mice were divided into three groups: Control (Sham), Model (TAC), and TAC mice treated with Ext.R (Ext.R), *n* = 8 for each group. B) Whole heart images and heart weight/tibia length ratio (HW/TL) of Sham, TAC, and TAC mice treated with Ext.R, *n* = 8 for each group. C) Serum NT‐proBNP levels were measured by ELISA, *n* = 8 for each group. D−F) Echocardiography assessments of cardiac function were performed in different groups. The calculation of cardiac function included: left ventricular (LV) mass index, interventricular septal thickness at systole and diastole (IVS; s, IVS; d), *n* = 8 for each group. G) Sections of hearts stained with Masson's trichrome to detect fibrosis (blue) and quantification of cardiac fibrosis in different groups of mice, *n* = 3 for each group. For each slice, 3 fields of view were randomly selected for analysis, Scale bar, 50 µm. H) Protein levels of fibrosis indices (*α*‐SMA and collagen‐3), antiapoptotic protein (Bcl‐xL), and antioxidant enzyme (SOD2) were determined by performing western blot analysis, *n* = 4 for each group. All data were analyzed using one‐way ANOVA and were expressed as means ± SD, #*p* < 0.05, ##*p* < 0.01 versus Sham group, *p < 0.05, **p < 0.01 versus TAC group.

It is unknown whether Ext.R could directly modulate cardiac hypertrophy. To address this question, we examined the effects of Ext.R on a mouse hypertrophy model subjected to transverse aortic constriction (TAC) for 28 days. Ext.R was administered to TAC mice on the second day after surgery, and maintained by one administration per day for 4 weeks (**Figure** **1**A). TAC induction resulted in significant cardiac hypertrophy compared to mice in the sham group, as estimated by heart weight to tibia length ratio (HW/TL). Ext.R treatment significantly reversed the change in HW/TL by TAC modeling (Figure 1B). The expression of N‐terminal prohormone of brain natriuretic peptide (NT‐proBNP), a diagnostic marker for left ventricular systolic dysfunction/heart failure, was also elevated in the TAC model group and later suppressed by Ext.R treatment (Figure 1C). Moreover, multiple cardiac function‐related echocardiographic parameters were significantly improved in the Ext.R‐treated group compared to the model group, including: left ventricular (LV) mass index, interventricular septal thickness at systole and diastole (IVS; s, IVS; d) (Figure 1D–F). Since interstitial fibrosis is a hallmark of maladaptive cardiac hypertrophy,^[^ ^19^ ^]^ we also analyzed the degree of myocardial fibrosis in the mice. Indeed, TAC surgery resulted in marked accumulation of fibrosis in the interstitial space, as well as increased expression of alpha‐smooth muscle actin (*α*‐SMA) and collagen‐3 (Col‐3) in cardiac tissues. These changes were all dramatically inhibited by Ext.R administration (Figure 1G,H). In addition, the expressions of antiapoptotic protein B‐cell lymphoma‐extra large (Bcl‐xL) and antioxidant enzyme superoxide dismutase‐2 (SOD2) were increased following Ext.R treatment, suggesting a potential antiapoptotic and antioxidant mechanisms of Ext.R‐mediated cardiac protection. Collectively, these results suggested that Ext.R was capable of blocking the cardiac hypertrophic response induced by TAC in mice.

We also treated Sham mice with saline or Ext.R, respectively. As shown in Figure S1 (Supporting Information), there is no effect of Ext.R on heart weight (Figure S1B, Supporting Information), cardiac function (Figure S1C,D, Supporting Information), cardiac histopathology (Figure S1E, Supporting Information), and the phosphorylation level of SGK1 (Figure S1F, Supporting Information) in Sham group. In addition, there were no significantly differences in serum level of regular biomarkers between Sham mice treated with saline and Ext.R. Interestingly, alkaline phosphatase (ALP) level was decreased by Ext.R treatment (Figure S2, Supporting Information), which needs to be further investigated.

### 2.2 Bioinformatic Profiling Revealed that Ext.R Regulated the Forkhead box protein O1 (FoxO1) Signaling Pathway and Identified SGK1 as a Potential Target

**Figure 2:**
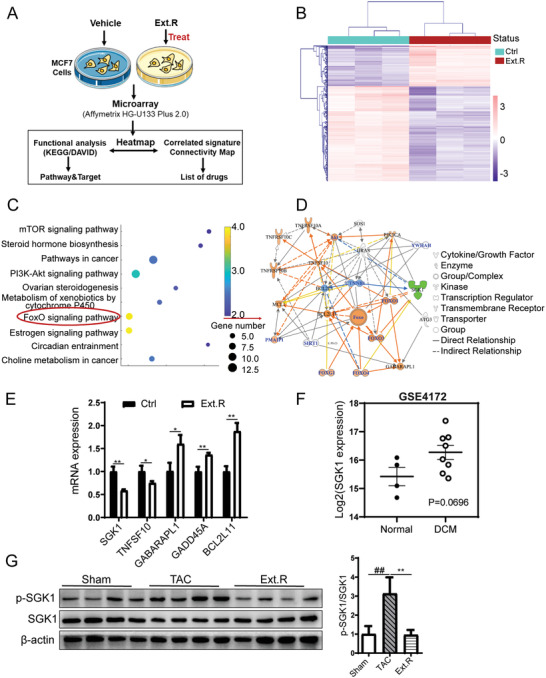
Quantification of transcriptome profiles in MCF‐7 cells treated with Ext.R. A) Overview of the strategy used in this study. A detailed description in the Experimental Section. Vehicle control (Ctrl) and Ext.R treated group (Ext.R), *n* = 3 independent experiments. B) The hierarchical clustering analysis and heatmap of the correlation coefficients between gene expression profiles. C) Top KEGG pathways enriched with DEGs. D) Visualization of PPI network related to FoxO signaling pathway. E) The representative genes in the FoxO signaling pathway were determined by RT‐PCR, *n* = 3 independent experiments. Data were analyzed using unpaired two‐tailed *t*‐test and were expressed as means ± SD, **p < 0.01, *p < 0.05 versus Ctrl group. F) SGK1 gene expression in the hearts of healthy patients and patients with dilated cardiomyopathy (DCM). The GEO accession number is GSE4172. Normal patients’ group: *n* = 4; DCM patients’ group: *n* = 8. Data were analyzed using unpaired two‐tailed *t*‐test, *p* = 0.0696. G) The phosphorylation level of SGK1 in myocardial tissues suffering from pressure overload, *n* = 4 for each group, data were analyzed using one‐way ANOVA and data were expressed as means ± SD, *##p *< 0.01 versus Sham group, **p < 0.01 versus TAC group.

To identify the downstream targets of Ext.R‐mediated cardiac protection, we leveraged the online Connectivity Map database (CMAP, Broad/MIT),^[^ ^20^ ^]^ which stores 1.5 million gene expression profiles derived from ≈5000 small‐molecule compounds and 3000 genetic reagents (http://www.broadinstitute.org/cMAP/). By comparing gene expression profile regulated by Ext.R with those of known compounds recorded in CMAP, we can speculate its possible pharmacological mechanism. Since most gene expression data in CMAP were derived from the breast cancer cell line (MCF‐7) or cervix cancer cell line (HeLa), we conducted microarray‐based transcriptional profiling using the MCF‐7 with or without Ext.R treatment (**Figure** **2**A). A total of 367 differentially expressed genes (DEGs) were identified (Table S1, Supporting Information), in which 109 DEGs were significantly up‐regulated (Figure 2B). These DEGs were subsequently converted to 102 up‐regulated and 224 down‐regulated probe sets and used as the query set for CMAP searching. Similar expression signatures between Ext.R and 37 drugs were identified, including cholinergic receptor, calcium receptor, and corticosteroid receptor blockers (Table S2, Supporting Information). Notably, Ext.R showed highest similarity to phenoxybenzamine, an alpha‐1A adrenergic antagonist,^[^ ^21^ ^]^ with a positive enrichment score of 1.0. Since adrenergic receptor antagonists are well‐known medicines for the treatment of cardiac hypertrophy and heart failure, we hypothesized that Ext.R may also possess adrenergic antagonist activity.

KEGG pathway analysis and Ingenuity Pathway Analysis (IPA) were also conducted to identify involved pathways. Among the top 10 impacted KEGG pathways (Figure 2C), the Forkhead box protein O (FoxO) pathway was of particular interest, given the involvement of FoxO1 in regulating energy homeostasis, oxidative stress response, and cell death, in addition to its crucial role in sustaining cardiomyocyte metabolism and cell survival.^[^ ^22^ ^]^ Furthermore, activation of FoxO1 by insulin resistance promotes metabolic dysregulation and heart failure.^[^ ^23^ ^]^ Therefore, we generated protein–protein interaction (PPI) network of DEGs in the FoxO signaling pathway to determine the molecules with the highest degree of protein interactions (Figure 2D). IPA functional analysis indicated that SGK1 acted as a hub of PPI network regulated by Ext.R, while significant activation of FoxO occurred after inhibiting SGK1 activity or its content (Figure S3, Supporting Information). SGK1 and tumor necrosis factor ligand superfamily member 10 (TNFSF10) were detected as the two major relevant signaling molecules downregulated by Ext.R in the FoxO pathway. Conversely, GABA type A receptor associated protein like 1 (GABARAPL1), growth arrest and DNA damage protein 45A (GADD45A) and Bcl‐2‐like 11 (BCL2L11) were upregulated by Ext.R. The expression changes of these five genes were further verified by qPCR (Figure 2E). Notably, SGK1 was previously related to the initiation of cardiac hypertrophy and remodeling.^[^ ^24^ ^]^ Specifically, it was reported that during cardiac hypertrophy, alpha‐adrenergic signaling was augmented by corticosteroid partially through SGK1 upregulation.^[^ ^25^ ^]^ After searching the Gene Expression Omnibus (GEO) database, we observed an increased trend of cardiac mRNA expression of SGK1 in patients with dilated cardiomyopathy (DCM) (GSE4172 dataset, Figure 2F), suggesting a potential role of SGK1 in the progression of heart hypertrophy. We therefore speculated that SGK1 may be an essential target for the cardioprotective effects of Ext.R. Supportively, a significant accumulation of SGK1 phosphorylation was detected in the heart tissue of TAC mice, which was largely blocked by Ext.R (Figure 2G). To validate the impact of Ext.R on FoxO1 signaling pathway, we further measured the FoxO1 and GSK3*β* protein levels in heart tissue of TAC model. The results showed that FoxO1 and glycogen synthase kinase‐3 beta (GSK3*β*) phosphorylation were significantly increased in TAC model as compared with Sham group, Ext.R treatment significantly decreased FoxO1 and GSK3*β* phosphorylation (Figure S4, Supporting Information). Taken together, our results suggested that anti‐SGK1 compounds in Ext.R may mediate its cardioprotective effects.

### 2.3 Screening SGK1 Inhibitors from Ext.R by a Mass Spectrometry‐Based Kinase Inhibitory Assay

**Figure 3:**
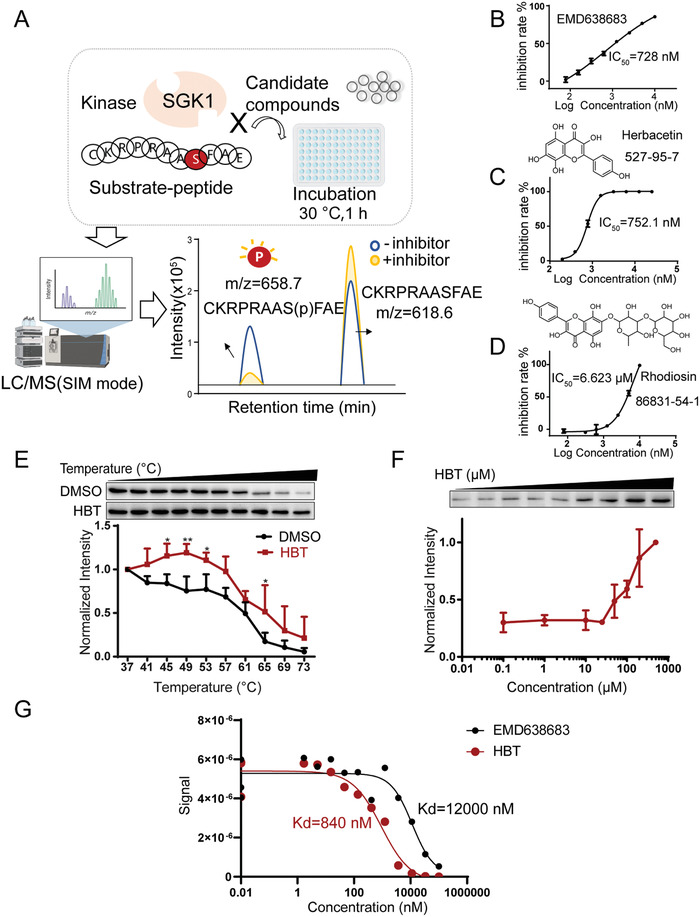
Mass spectrometry‐based kinase inhibitory assay for screening SGK1 inhibitor. A) Illustration of the mass spectrometry‐based assay for screening SGK1 inhibitors. B) IC_50_ values of EMD638683 (positive control) to SGK1 activity, *n* = 3 independent experiments. C,D) Structure and IC_50_ values of HBT and Rhodiosin to SGK1 activity, *n* = 3 independent experiments. E) HBT treatment (100 µmol) increases the thermal stability of SGK1 in cell lysates as measured by a temperature‐dependent cellular thermal shift assay, *n* = 4 independent experiments. F) HBT treatment increases the thermal stability of SGK1 in cell lysates as measured by a concentration‐dependent cellular thermal shift assay at 65 °C, *n* = 3 independent experiments. G) Direct binding assay of HBT to SGK1 by KINOMEscan assay (Eurofins Discovery).

To identify active compounds with SGK1 inhibitory activity from Ext.R, a novel mass‐spectrometry based kinase inhibitory assay was developed. A polypeptide sequence (CKRPRAASFAE, m/z: 618.6) derived from glycogen Synthase Kinase‐3(GSK3), a known substrate of SGK1, was optimized and synthesized. We subsequently used liquid chromatography coupled with mass spectrometry (LC/MS) to detect and quantify its phosphorylation level (CKRPRAAS(p)FAE, m/z:658.7) (**Figure** **3**A).^[^ ^26^ ^]^ Basal level SGK1 was supplemented to each well, and the peak area of phosphorylation was measured in the presence or absence of testing compounds. As shown in Figure S5A (Supporting Information), the peak area was steadily increased with an increasing concentration of SGK1, and exhibited a positive linear relationship with the elevation of SGK1 concentration from 0.05 to 0.3 µg mL^−1^ (*R* ^2^ = 0.9945). The assay also exhibited satisfying intraday stability (Figure S5B, Supporting Information). To validate the sensitivity of MS‐based assay, the SGK1 inhibitor EMD638683 was used as a positive control. As shown in Figure 3B, the IC_50_ value of EMD638683 closely matched previous findings at 728 nmol, thereby confirming the validity of the assay.^[^ ^27^ ^]^ We then used the assay to verify the inhibitory effects of Ext.R on SGK1 activity and observed an IC_50_ value of 440 ng/mL (Figure S5C, Supporting Information). After investigating the major chemical constituents in Ext.R (Table S3, Supporting Information), a set of compounds in Ext.R were subsequently screened for their impacts on SGK1 activity. Three SGK1 inhibitors were found, including herbacetin (HBT), rhodiosin, and p‐coumaric acid, with IC_50_ values of 752.1 nmol, 6.623 µmol, and 8.036 µmol (Figure 3C,D; and Figure S5D, Supporting Information). Since HBT displayed the best activity among those compounds, it was chosen as the representative compound for further study. The direct interaction between HBT and SGK1 was further verified by temperature‐ and dose‐dependent cellular thermal shift assays (Figure 3E,F). We further performed a direct binding assay of HBT to SGK1 by KINOMEscan assay (Eurofins Discovery). The binding constants (Kds) of EMD638683, a known inhibitor of SGK1 was 12 000 nmol. Interestingly, the Kds value of HBT for SGK1 was 840 nmol, which is 14‐fold lower than EMD638683 in the same assay (Figure 3G). Those results indicated that HBT is a promising lead compound as SGK1 inhibitor.

### 2.4 D177 of SGK1 is Required for Its Interaction with Herbacetin

**Figure 4:**
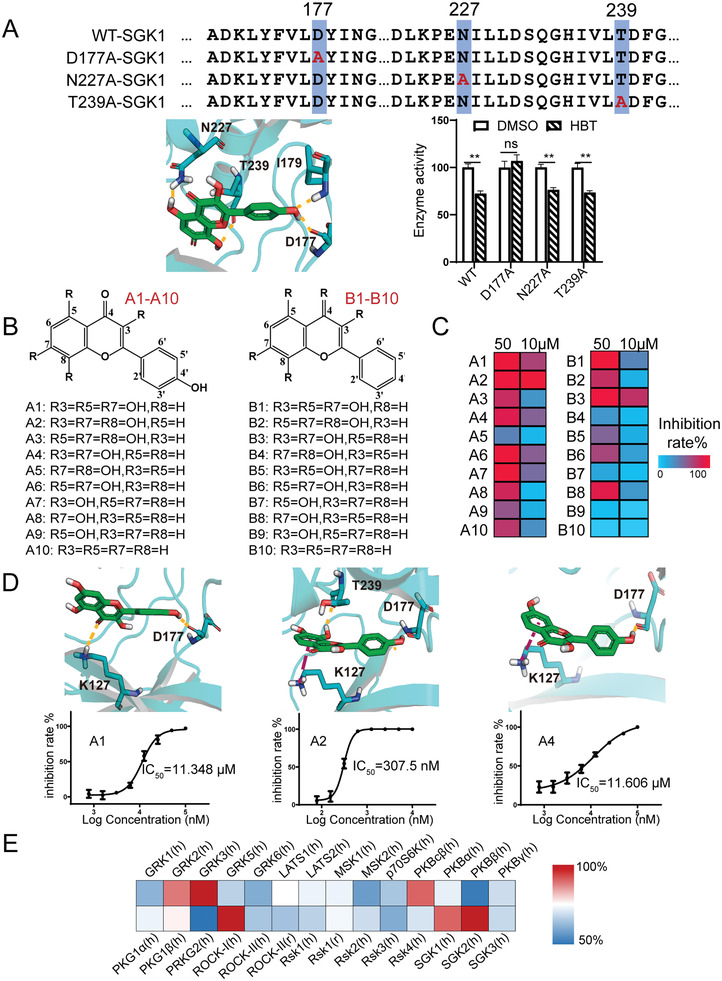
A) Structure of the SGK1 complex docking with HBT. Mutations of predicted key residues abolished SGK1 inhibition by HBT, *n* = 3 independent experiments. Statistical analysis was performed using unpaired two‐tailed *t*‐test and data were expressed as means ± SD, **p < 0.01 versus vehicle control (DMSO), ns: no significant. The molecule and the neighboring SGK1 residues are shown in detail. The inhibition rates of HBT toward SGK1 mutations were normalized to WT SGK1. B) Chemical structures of the structurally‐related flavonoids. C) Inhibitory activity of flavonoids in A and B classes against SGK1. D) Predominant interactions and IC_50_ values of representative flavonoids against SGK1, *n* = 3 independent experiments. E) Inhibitory effects of HBT on 28 human AGC kinases tested by KinaseProfiler Assay.

To further elaborate the interaction between SGK1 and HBT, the SGK1‐HBT complex was constructed by molecular docking based on the SGK1 crystal structures retrieved from the Protein Data Bank (PDB). According to the consistency between the IC_50_ values and predicted binding scores for the hit compounds of Ext.R and EMD638683 (Table S4, Supporting Information), the crystal structure of 3HDN^[^ ^28^ ^]^ was selected as the template to predict the binding structure of HBT. As shown in **Figure** **4**A, the key residues D177, N227, and T239 were suggested to be essential for the binding of HBT. To explore the impacts of these three residues on the SGK1‐HBT binding, plasmids carrying respective SGK1 mutations (D177A, N227A, and T239A) were designed and transfected in the human embryonic kidney cells (HEK293T). The FLAG‐tagged wild‐type or mutated SGK1 protein lysates were collected by immunoprecipitation and examined for enzymatic activity in the presence of HBT using MS‐based kinase inhibitory assay. SGK1 protein with the D177A mutation almost completely lost its response to HBT. Thus, these results indicated that the D177 residue of SGK1 was indispensable for the binding between HBT and SGK1.

### 2.5 HBT Hydroxyl Group Number and Position are Critical for Its Interaction with SGK1

HBT and rhodiosin share the same flavone pharmacophore, suggesting it is a key structural feature for SGK1 binding. 20 structurally related flavonoids of HBT were collected, which differ in the number and position of —OH groups, and examined for their effects on SGK1 activity. Based on the docking prediction, the —OH group at the C‐4’ position of HBT was required for the interaction with the key SGK1 residue D177. Therefore, the 20 flavonoids were divided into two classes according to the presence or absence of an —OH group at C‐4’ (Figure 4B). At both tested concentrations, flavonoids with an —OH group at C‐4’ (class A) inhibited SGK1 more than those without an —OH group at C‐4’ (class B) (Figure 4C). The IC_50_ of each compound is listed in Figure 4D; and Figure S6 (Supporting Information). Among all the tested compounds, A2 (3,7,8‐trihydroxy‐2‐(4‐hydroxyphenyl) chromen‐4‐one), which lacks an —OH group at C‐5 compared to HBT, showed the highest SGK1 inhibitory activity with a IC_50_ of 307.5 nmol. Conversely, the absence of an ‐OH group at C‐8 led to a greater than 10‐fold reduction in flavonoid SGK1 inhibitory activity, as shown by the performance of A1 or A4. The absence of an —OH group at C‐3 (A3 and A5) also led to a significant reduction in SGK1 inhibitory activity. Taken together, these results indicated that the presence of —OH groups at the C‐3, C‐8, C‐4’ positions was required for SGK1 interaction with HBT derivatives, whereas the absence of an —OH group at the C‐5 position may further enhance SGK1 inhibitory activity. The inhibitory activity of HBT (10 µmol) against 28 human AGC kinases was tested by KinaseProfiler Assay (Eurofins Discovery). The percentage of HBT's inhibitory activity against each kinase was normalized by the respective positive inhibitors of AGC kinases. As shown in Figure 4E, HBT exhibited selective inhibitory activity on SGK1 (95%) and SGK2 (105%) compared to SGK3 (77%) and cGMP‐dependent type II protein kinase (PRKG2) (45%) among AGC family kinases. We also observed high inhibitory effects of HBT for G protein‐coupled receptor kinase 3 (GRK3), Rho‐associated protein kinas‐1 (ROCK‐I), and the catalytic subunit C‐beta of PKA (PKAc*β*), which are warranted to further investigation.

### 2.6 HBT Blocks Cardiomyocyte Hypertrophy and FoxO1 Phosphorylation In Vitro

**Figure 5:**
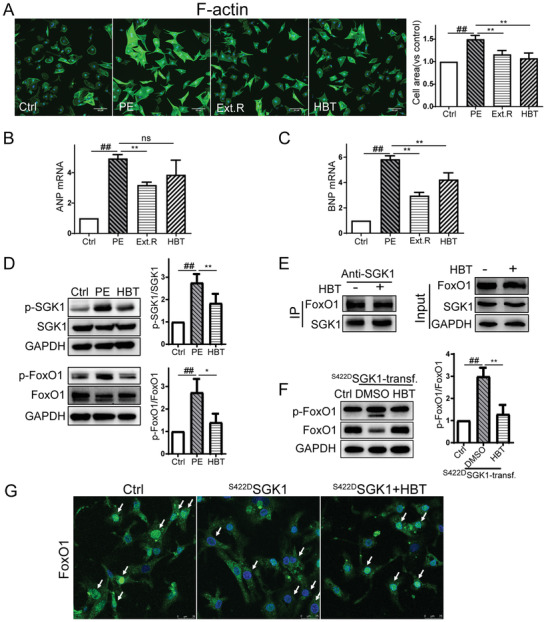
HBT blocks induction of cardiac hypertrophic responses and the SGK1/FoxO1 signaling pathway. A) Cardiomyocytes were treated with 50 µmol PE in the presence or absence of Ext.R (50 µg mL^−1^) or HBT (50 µmol) for 48 h. Cardiomyocytes were identified by cytoskeleton staining through the binding of phalloidin to F‐actin (green). Images were acquired using an ImageXpress Micro Confocal High‐Content Imaging System (Molecular Devices), with a 40 × PlanFluor objective. Relative cell area was quantified by the analysis module of MedExpress High‐Content Image and Analysis Software (Molecular Devices). Control group (Ctrl), PE group, PE plus Ext.R group (Ext.R), and PE plus HBT group (HBT), *n* = 3 independent experiments, Scale bar, 50 µm. B,C) Quantification of relative ANP and BNP in cardiomyocytes treated with Ext.R or HBT, *n* = 3 independent experiments. D) Western blot analysis of related proteins in PE‐treated cardiomyocytes, *n* = 3–4 independent experiments. E) Co‐IP and western blot analysis of SGK1‐FoxO1 interaction in cardiomyocytes, *n* = 3 independent experiments. F) Western blot analysis of related proteins in nontransfected (control adenovirus) and in ^S422D^SGK1‐transfected primary cardiac myocytes treated with DMSO as solvent control (^S422D^SGK1‐transf. group) or HBT (50 µmol, ^S422D^SGK1‐transf.+HBT group), *n* = 3 independent experiments. G) Confocal microscopy of nuclear translocation of FoxO1 in nontransfected (control adenovirus), ^S422D^SGK1‐transfected cardiomyocytes treated with HBT (50 µmol) or DMSO as solvent control. Green signal represents FoxO1 and white arrow represents the translocation of FoxO1 to nuclei, *n* = 3 independent experiments, Scale bar, 25 µm. All data were analyzed using one‐way ANOVA and data were expressed as means ± SD, *##p *< 0.01 versus Ctrl group, *p < 0.05, **p < 0.01 versus PE group or ^S422D^SGK1‐transf. group.

We further validated the antihypertrophy effects of HBT in primary rat cardiomyocytes treated with phenylephrine (PE), a widely‐used cardiac hypertrophic stimulant. The magnitude of cardiac hypertrophy was then evaluated by directly measuring the cell area after Alexa Fluor 488 phalloidin staining. As shown in Figure S7 (Supporting Information), antihypertrophy activity of compounds in Ext.R was evaluated by high‐content screening system. As a result, only HBT (50 µmol) was identified to attenuate PE‐induced hypertrophy, while compounds with poor anti‐SGK1 activity are ineffective in vitro. Therefore, we continued to investigate the pharmacological mechanism of HBT against cardiac hypertrophy. As shown in **Figure** **5**A, area of cardiomyocytes were increased after PE stimulation, either Ext.R or HBT can inhibit the phenotypic change. Meanwhile, significantly increased transcriptional level of atrial natriuretic peptides (ANP) and brain natriuretic peptide (BNP) (Figure 5B,C) were inhibited by Ext.R or HBT treatment. The protein expression of skeletal muscle α‐actin (ACTA1), BNP, and Myosin were also greatly increased after PE stimulation, and then significantly reduced by HBT treatment (Figure S8, Supporting Information). Importantly, the expression of phosphorylated‐SGK1 (p‐SGK1) and its downstream target p‐FoxO1 were greatly enhanced after PE stimulation. HBT treatment can significantly reduce the phosphorylation of SGK1 and FoxO1 (Figure 5D).

Since previous studies suggested that FoxO1 phosphorylation promotes its nuclear exclusion, whereas SGK1 binds and phosphorylates FoxO1, which thereby alters the subcellular localization of FoxO1 and leads to its transcriptional inactivation.^[^ ^29^ ^]^ In the present study, we studied HBT's effect on protein binding between SGK1 and FoxO1 by coimmunoprecipitation (Figure 5E). Surprisingly, we did not detect significant impact of HBT on the direct binding between SGK1 and FoxO1. Nevertheless, as previously reported,^[^ ^30^ ^]^ FoxO1 phosphorylation was significantly increased in cardiomyocytes after expression of the constitutively active SGK1 mutant SGK1(S422D), and HBT treatment was able to reverse the alternation (Figure 5F). Supportively, after transfection with the SGK1(S422D) plasmid, we consistently detected increased cytoplasmic signal of FoxO1 compared to nontransfected cells in which the majority of FoxO1 signal was distributed in the nucleus. Treatment with HBT was sufficient to restrain FoxO1 in the nucleus, suggesting reduced phosphorylation (Figure 5G). These results indicated that HBT may modify the activity of SGK1 rather than affecting its binding to protein targets.

### 2.7 HBT Downregulated PE‐Induced Oxidative Stress and Calcium Accumulation in Rat Cardiomyocytes

**Figure 6:**
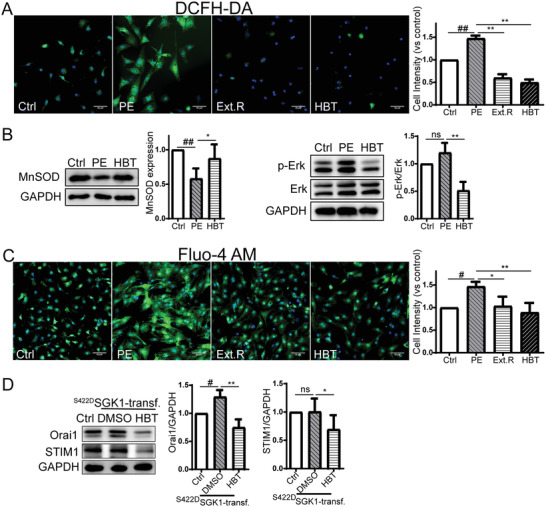
HBT alleviates oxidative damage and calcium accumulation in the development of cardiac hypertrophy. A) Fluorescent microscopy of cardiomyocytes stained with H_2_DCFDA, *n* = 4 independent experiments, Scale bar, 50 µm. B) Western blot analysis of MnSOD and Erk expression in PE‐treated cardiomyocytes, *n* = 3–4 independent experiments. C) Fluorescent microscopy of cardiomyocytes stained with a Fluo4‐AM probe, *n* = 3 independent experiments, Scale bar, 50 µm. D) Western blot analysis of Orai1 and STIM1 in ^S422D^SGK1‐transfected primary cardiac myocytes treated with HBT (50 µmol) or DMSO as solvent control, *n* = 3–5 independent experiments. All data were analyzed using one‐way ANOVA and data were expressed as means ± SD, *#p *< 0.05, *##p *< 0.01 versus Ctrl group, *p < 0.05, **p < 0.01 versus PE group or ^S422D^SGK1‐transf. group.

Since increased oxidative stress and cytoplasmic calcium were suggested to be two key pathological mechanisms of cardiomyocytes hypertrophy,^[^ ^7^, ^8^ ^]^ we examined the effects of Ext.R and HBT on these processes. After PE stimulation, an elevated level of free radicals was observed in rat cardiomyocytes, as shown by staining with the ROS probe 2', 7'‐dichlorodihydrofluorescein diacetate (DCFH‐DA). Cotreatment with Ext.R or HBT greatly inhibited this increase in oxidation (**Figure** **6**A). Meanwhile, the expression of the mitochondrial antioxidant manganese superoxide dismutase (MnSOD) was also inhibited by PE and restored when HBT was administrated concomitantly. Moreover, HBT downregulated the phosphorylation level of extracellular signal‐regulated kinase (Erk), which is usually activated in oxidative stress signaling (Figure 6B).^[^ ^31^, ^32^ ^]^ In summary, these results suggested that HBT was capable of reducing oxidative stress levels in cardiomyocytes upon hypertrophic stimulations.

Elevated cytoplasmic calcium was also observed in PE‐stimulated rat cardiomyocytes after Fluo‐4 AM staining. Cotreatment with Ext.R or HBT effectively blocked calcium accumulation (Figure 6C). Since the channel subunit Orai1 and stromal interaction molecule 1 (STIM1) are known to participate in the orchestration of Ca^2+^ entry into cells and are regulated by multiple kinases including SGK1,^[^ ^33^ ^]^ we examined if HBT regulates their activities. In cells with constitutively expressed SGK1, an increased level of Orai1 was detected, which was significantly inhibited by HBT. HBT also moderately inhibited the expression of STIM1; however, STIM1 expression was not upregulated by SGK1 overexpression (Figure 6D). Orai1 may be regulated by Nedd4‐like E3 ubiquitin protein ligase (Nedd4l) mediated degradation or the transcription factor nuclear factor‐κB (NF‐*κ*B) dependent transcription, so we detected the effects of HBT on SGK1 downstream proteins. We found that HBT could reduce the phosphorylation of Nedd4l induced by PE and the phosphorylation of N‐myc downstream regulated gene 1 (NDRG1) in primary cardiomyocytes transfected with continuously‐activated form of SGK1, but it did not affect the phosphorylation level of NF‐*κ*B p65 (Ser536) (Figure S9, Supporting Information). Therefore, HBT may also attenuate cardiomyocyte hypertrophy by reducing cytoplasmic calcium level.

### 2.8 HBT Inhibits ISO‐Induced Cardiac Hypertrophy in Mice

**Figure 7:**
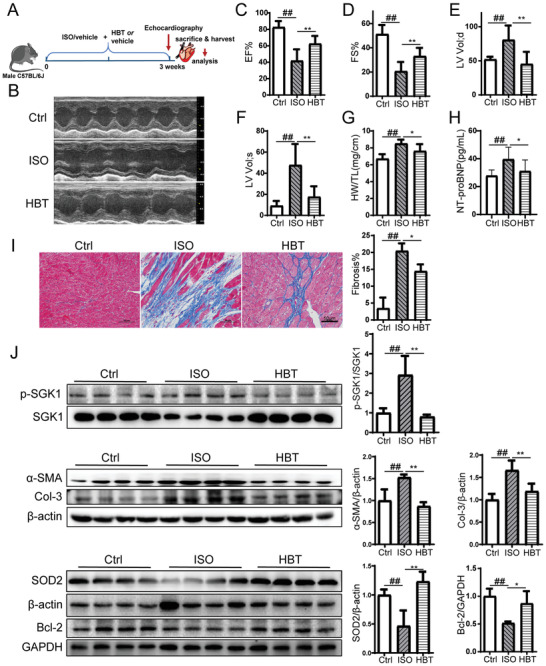
HBT protects mice from an ISO‐induced cardiac hypertrophic response. A) Schedule of animal treatments. 5 mg kg^−1^ d^−1^ ISO was given for 21 days by subcutaneous injection. The mice were divided into three groups: control (Ctrl), ISO alone (ISO) and ISO + HBT (HBT), *n* = 9 for each group. B–F) Echocardiography assessments of cardiac function were performed in different groups, *n* = 9 for each group. G) The heart weight/tibial length (HW/TL) of control, ISO, and ISO mice treated with HBT, *n* = 9 for each group. H) Serum NT‐proBNP levels were measured by ELISA, *n* = 9 for each group. I) Sections of hearts stained with Masson's trichrome to detect fibrosis (blue) and quantification of cardiac fibrosis in different groups of mice, *n* = 3 for each group. For each slice, 3 fields of view were randomly selected for analysis, Scale bar, 50 µm. J) Tissue lysates were prepared and analyzed by western blotting with the indicated antibodies, *n* = 4 for each group. All data were analyzed using one‐way ANOVA and data were expressed as means ± SD, *#p *< 0.05, *##p *< 0.01 versus Ctrl group, *p < 0.05, **p < 0.01 versus ISO group.

Finally, we assessed the cardioprotective effect of HBT in mice infused with the hypertrophic agonist isoproterenol (ISO). Mice were challenged with ISO for 21 days via subcutaneous injection and then were administered with 10 mg kg^−1^ of HBT once per day until the mice were sacrificed (**Figure** **7**A). HBT administration effectively improved cardiac performance in ISO mice. Specifically, HBT treatment resulted in increased left ventricular ejection fraction (EF%), left ventricular fraction shortening (FS%), as well as decreased left ventricular volume at both the end‐diastole and end‐systole stages (LV Vol; d and LV Vol; s) (Figure 7B–F). After ISO stimulation, the heart weight/tibial length (HW/TL) was significantly increased over the control mice, indicating the development of heart hypertrophy. HBT administration was sufficient to reduce the HW/TL level (Figure 7G). Moreover, HBT‐treated mice showed a decreased level in the NT‐proBNP reduction response, the gold standard biomarker of heart failure (Figure 7H).^[^ ^34^ ^]^

The accumulation of collagen fibrosis was also reduced by HBT in ISO‐treated mice as revealed by Masson's staining (Figure 7I). Myocardial interstitial fibrosis is maladaptive and contributes to left ventricular dysfunction, impaired myocardial oxygen availability, poor outcomes, and leads to the development of pathological ventricular remodeling. We found that HBT reduced expression of p‐SGK1 and fibrosis markers *α*‐SMA and Col‐3 in ISO mice (Figure 7J), thereby suggesting that HBT was able to block hypertrophic responses. Immunofluorescent staining revealed a similar result for the transforming growth factor‐β (TGF‐*β*)‐induced cardiomyocyte fibrosis (Figure S10, Supporting Information). Furthermore, decreased levels of the antiapoptotic protein Bcl‐2, and decreased MnSOD levels were detected in ISO mice. These changes were almost completely reversed by HBT.

The protein expression of phos‐FoxO1 and other SGK1 down‐streaming proteins were analyzed in the heart tissue of ISO mouse model. As shown in Figure S11 (Supporting Information), HBT significantly decreased FoxO1 phosphorylation, as well as the protein expression of calcium accumulation related proteins STIM1 and Orai1. Moreover, HBT increased the level of oxidative stress related protein heme oxygenase‐1 (HO‐1) and nuclear factor erythroid 2‐related factor 2 (Nrf2).

Finally, we evaluate the safety and pharmacokinetics of HBT to ensure its druggability. The result showed that there is no adverse effect of HBT on heart weight, cardiac function, or cardiac histopathology of control mice (Figure S12, Supporting Information). In addition, there was no significant difference in the serum level of regular biomarkers such as alaninetransaminase (ALT), aspartate aminotransferase (AST), and total cholesterol (TCHO) between control mice and HBT‐treated mice (Figure S13, Supporting Information). Single dose administration of HBT at 40 mg kg^−1^ was uptaken rapidly with a maximal plasma concentration of ≈6.73 ng mL^−1^ after 10 min (Figure S14A, Supporting Information). At this timepoint, HBT can be readily detected in heart, liver, and kidney (Figure S14B, Supporting Information). These findings suggested that HBT can be efficiently absorbed and was distributed into heart and other tissues to exert pharmacological effects.

In summary, our findings demonstrated the in vivo effects of HBT in inhibiting cardiac hypertrophic responses and promoting cardiac function in mice.

## 3 Discussion

In this study, we identified an active compound from Ext.R that alleviated cardiac hypertrophic responses in mice and identified its protein target through in vitro and in vivo analysis. HBT was identified as a novel SGK1 inhibitor that directly binds SGK1 and reduces its phosphorylation level. Subsequently, decreased phosphorylation and nuclear exclusion of FoxO1 may regulate heart function through downstream oxidative stress signaling and calcium release.

Ext.R is a botanical adaptogen found worldwide with various protective effects on neurodegenerative diseases, cardiovascular diseases, diabetes, aging, and cancer. It was previously reported that Ext.R administration could reverse left ventricular electrical remodeling and attenuate atrial fibrosis in rabbits with coronary ligation‐induced heart failure, possibly via ion channel regulation and activation of PI3K/AKT signaling.^[^ ^35^ ^]^ In our study, we identified HBT, a less studied compound of Ext.R, as a key SGK1 inhibitor essential for the herb's cardiac protective function. HBT was previously reported to possess antitumor, antioxidation, and anti‐inflammation roles. HBT has also been suggested to be an allosteric inhibitor of ornithine decarboxylase, with antitumor activity in a preclinical model of colon cancer.^[^ ^36^ ^]^

Through transcriptomics‐based KEGG pathway analysis, we speculated that SGK1 was a key factor mediating the regulatory function of Ext.R in cardiac hypertrophy. As a serine/threonine protein kinase, SGK1 is structurally close to AKT. The catalytic domain of SGK1 is 54% homologous to AKT and shares functional similarities with the AKT family of kinases.^[^ ^37^ ^]^ Therefore, the PKB/Akt peptide substrate (CKRPRAASFAE) derived from GSK‐3*β* was selected as a mass spectrometry probe in an SGK1 inhibitor screening assay. We identified three SGK1 inhibitors, including: p‐coumaric acid, HBT, and HBT derivative rhodiosin from Ext.R. HBT had the best inhibitory activity toward SGK1, and HBT was found to directly bind to SGK1. Mechanistic investigations determined that Asp177 in SGK1 was a critical site for binding. Furthermore, we investigated whether the hydroxyl (OH) residues of HBT were involved in its inhibition of SGK1. We tested 20 flavonoids structurally similar to HBT using an in vitro SGK1 activity assay and identified the most important structural features for SGK1 inhibition. Another six flavonoids were found to display SGK1 inhibitory activity.

Several SGK1 inhibitors have been reported previously. The effectiveness of HBT was comparable with existing SGK1 inhibitors. An IC_50_ of 62 nmol was reported for GSK650394 (GSK), based on the scintillation proximity assay of SGK1 activity;^[^ ^38^ ^]^ whereas an IC_50_ of 3.35 µmol was reported for EMD638683 (Merck) regarding to SGK1‐dependent phosphorylation of NDRG1.^[^ ^27^ ^]^ In our study, the IC_50_ of HBT was 752 nmol based on the MS‐based SGK1 kinase inhibitory assay, suggesting that HBT possesses potent kinase activity which is comparable to representative SGK1 inhibitors. Moreover, HBT treatment of 10 mg kg^−1^ d^−1^ for 21 days is sufficient to alleviate mice cardiac hypertrophy without causing any significant toxicity, demonstrating the high safety and efficacy of the compound. We further calculated the structural similarity of HBT and 9 reported SGK1 inhibitors. As shown in Figure S15 (Supporting Information), the skeleton and structure of HBT is quite different from previously reported compounds. Taken together, these data suggested that HBT is a promising novel drug candidate of SGK1 inhibitor in the management of cardiomyocyte hypertrophy.

Enhanced SGK1 expression and/or kinase activity have been linked to several diseases including: cardiac fibrosis, hypertension, diabetes, diabetic nephropathy, and the development of several human tumors. Previous studies suggested that SGK1 is an antihypertrophic molecule whose deficiency counteracts cardiac remodeling during pressure overload, glucocorticoid excess, and angiotensin II infusion.^[^ ^39^ ^]^ It is also involved in complex intracellular signaling through fibrotic, inflammatory, and oxidative pathways, which are essential for cardiac hypertrophy progression.^[^ ^24^ ^]^ SGK1 directly phosphorylates many downstream targets, including: GSK3*β*, FoxO3a, and FoxO1. During cardiac hypertrophy, SGK1 also fosters the cytoplasmic localization of cyclin‐dependent kinase inhibitor 1B (p27), a protein which suppresses cardiac hypertrophy.^[^ ^29^, ^40^ ^]^ Taken together, these findings suggest that SGK1 is a key factor in the progress of cardiac hypertrophy and thus a promising therapeutic target.

SGK1 binds to and phosphorylates FoxO1.^[^ ^29^ ^]^ Consistent with previous results, we found that the increase in SGK1 phosphorylation in PE‐induced hypertrophic cardiomyocytes closely paralleled the increase in FoxO1 phosphorylation. The interaction between SGK1 and FoxO1 could also be detected in primary myocardial cells of neonatal rats. Phosphorylated FoxO1 is inactivated and localized to the cytoplasm, thereby losing its ability to regulate downstream targets. In contrast, activation of FoxO1 transcription factor enhances oxidative stress resistance through up‐regulation of two antioxidant enzymes, catalase, and MnSOD, both known to be downstream targets of FoxO1.^[^ ^41^ ^]^ Supportively, we observed increased FoxO1 nuclear retention and MnSOD expression in HBT treated cardiomyocytes. Downregulated Erk1/2 signaling, which is a well‐characterized ROS‐sensitive signaling pathway in the progression of cardiac hypertrophy,^[^ ^42^ ^]^ was also detected in HBT supplemented cells. Therefore, HBT may regulate cardiac hypertrophy by mediating FoxO1‐dependent oxidative stress signaling.

Additionally, we detected increased cytoplasmic calcium in hypertrophic cardiomyocytes by Fluo‐4 AM staining. The protein abundance of Orai1 was also significantly higher in SGK1(S422D) cardiomyocytes. Orai1 is a plasma membrane protein regulated by STIM1 and acts as the pore‐forming unit for store‐operated Ca^2+^entry (SOCE). Upregulated Orai1 was reported to be a stress response molecule in cardiac hypertrophy, and Orai1 channel inhibition preserved left ventricular systolic function and promoted normal Ca^2+^ handling after pressure overload.^[^ ^8^ ^]^ A regulatory role of SGK1 on the expression of Orai1/STIM1 was suggested by previous studies.^[^ ^43^, ^44^ ^]^ Consistent with this, decreased calcium accumulation and Orail1/STIM1 were detected in HBT‐treated cardiomyocytes, suggesting that HBT‐mediated heart protection may be related to its regulation of calcium levels via inhibiting SGK1. Previous studies further supported that FoxO1 activation regulates calcium homeostasis and represses cardiac hypertrophy by inhibiting calcineurin, which is directly regulated by calcium.^[^ ^45^ ^]^ However, there is no direct evidence of a relationship between FoxO1 and Orai1.

We further confirmed our in vitro results in vivo. Mice treated with HBT developed significantly less fibrosis, along with smaller heart sizes, and improved cardiac function when subjected to isoproterenol infusion compared to untreated mice. We further found that HBT treatment prevented cells from apoptosis and oxidative stress. In sum, these results suggested that inhibition of SGK1 by HBT could be a potential therapeutic strategy to prevent cardiac remodeling.

Three limitations need to be addressed in our current study. First, different cardiac hypertrophy models were employed to study the effects of the Ext.R (TAC model) and HBT (ISO model) in our study. Although we believed that either model is sufficient to support the antihypertrophic effect of the herb and its active compound, since both of them are widely reported cardiac hypertrophy models, the pathological mechanisms varied. Therefore, future pharmacodynamics and pharmacological studies in both TAC and ISO models will be valuable to further validate the drug effects. Second, the 28‐panel kinase assay suggested that HBT showed effective inhibitory activity against not only SGK1 but also other AGC kinases, such as SGK2 and GRK3, which may be connected with off‐target effects of HBT. A rationally structure optimization of HBT retaining high on‐target activity while reducing off‐target effects is warranted to further investigation. Third, the single dose PK and distribution data suggested HBT can be absorbed and was distributed into heart and other tissues to exert pharmacological effects. As a SGK1 inhibitor derived from natural sources, the potency and ADME properties of HBT should be further optimized by pharmacochemical strategies to increase its potency and specificity in targeting SGK1.

## 4 Conclusion

In this study, we demonstrated the anticardiac hypertrophy effect of Ext.R, and identified its major active component as HBT, which possibly protects heart function through inhibiting SGK1 activity. Our study also illustrates a mass spectrometry‐based strategy to identify novel SGK1 inhibitors from complex mixture of traditional Chinese medicine. Using this strategy, we identified HBT as a novel inhibitor of SGK1, and we showed that HBT was capable of blocking the progress of cardiac hypertrophy both in vitro and in vivo. Moreover, a panel of nine flavonoids with SGK1 inhibitory activities was proposed, which can be used as valuable leads for the search and design of new therapeutics for cardiac hypertrophy as well as other SGK1‐driven diseases.

## 5 Experimental Section

### Chemicals and Reagents

Ext.R was supplied by Tonghua Yusheng Pharmaceutical Co. Ltd. (Tonghua, China). Representative LC‐MS chromatograms of *Rhodiola wallichiana var.cholaensis* and *Rhodiola crenulata* were shown in Figure S16 (Supporting Information). Herbacetin (HBT) and Rhodiosin were purchased from Shanghai Yuanye Bio‐Technology Co., Ltd (Shanghai, China). EMD638683 was purchased from MCE (HY‐15193, MCE, China). HPLC‐grade acetonitrile and methanol were obtained from Fisher (Leicestershire, UK). Ultrapure water was obtained from a Milli‐Q academic ultrapure water system (Millipore, Milford, MA). LC‐MS/HPLC‐grade formic acid (FA) was obtained from Anaqua Chemicals Supply (Wilmington, USA).

### Mice

Male C57BL/6J mice were obtained from Shanghai Slac Laboratory Animal Technology Co., China. All animals were fed a standard laboratory diet and housed on a 12 h light/dark cycle at 25 ℃ with unrestricted access to food and water for the duration of the experiment. All animal studies were performed in accordance with the Institutional Animal Care and Use Committee of Zhejiang University and the Guide for the Care and Use of Laboratory Animals published by the US National Institutes of Health (NIH Publication No. 85‐23, revised 1996).

For transverse aortic constriction (TAC)‐mediated cardiac hypertrophy, aortic banding was performed in 6‐week‐old adult male mice to produce pressure‐overload hypertrophy. A total of 24 mice were randomly divided into 3 groups, control group (Sham), TAC group, and TAC plus Ext.R group (Ext.R), *n* = 8 for each group. Mice were anaesthetized via intraperitoneal injection of 80 mg kg^−1^ pentobarbital sodium. The chest was opened to expose the aorta as identified between the origin of the innominate and left common carotid arteries. A 4‐0 silk suture was ligatured around the aorta over a 26‐gauge needle (in order to keep the models consistent) and the needle was subsequently removed followed by closing the incision on the chest. Animals with sham surgery underwent an identical surgical procedure but without band placement. Ext.R was injected at a dose of 10 mL kg^−1^ day^−1^ intraperitoneally followed by echocardiography measurement at day 28. Mice in Sham and TAC groups were injected with the same amount of solvent.

For isoproterenol (ISO)‐mediated cardiac hypertrophy, mice were randomly divided into 3 groups, control group (Ctrl), ISO group and ISO plus HBT group (HBT), 5 mg kg^−1^ day^−1^ ISO was given for 21 days by subcutaneous injection. The control mice were treated with 1% DMSO‐vehicle. At the same time, male C57BL/6 mice in HBT group were intraperitoneally injected with 10 mg kg^−1^ d^−1^ HBT diluted with a solution containing 1% DMSO and 99% saline. Echocardiography was performed on conscious mice using a Vevo 2100 system. M‐mode images of the left ventricular short axis were captured. After echocardiography evaluation, the mice were anesthetized via intraperitoneal injection of 240 mg kg^−1^ pentobarbital sodium and were euthanized by excising the heart rapidly.

### Microarray Processing and Data Analysis

Gene profiling was performed by Shanghai OE Biotech CO., Ltd, using an Affymetrix Human Genome U133 Plus 2.0 array (Affymetrix; Thermo Fisher Scientific Inc., USA). Briefly, 1×10^6^ MCF‐7 cells (a human breast cancer cell line) were seeded in 60×15 mm plates and incubated at 37 °C in a 5% CO_2_ atmosphere. After 24 h, Ext.R was added to the plate and incubated for another 6 h at 37 °C. Total RNA was isolated from MCF‐7 cells using Trizol reagent (CWBIO, China) according to the manufacturer's protocol and amplified, labeled, and purified using GeneChip IVT Express Kits (Affymetrix, 901 229) following the manufacturer's instructions to obtain biotin labeled cRNA. Then, the GeneChip Hybridization, Wash, and Stain Kit (Affymetrix, 900 720) was used following the manufacturer's instructions. Slides were scanned by a GeneChip Scanner 3000 (Affymetrix) and a Affymetrix GeneChip Command Console 4.0 with default settings. The raw microarray data are available through the GEO database (GEO series accession number: GSE166975).

Genes were considered differentially expressed if the fold changes were ≥ 2, and the corrected *P* value were less than 0.05. Heatmaps were generated using Multi Experiment Viewer software (MEV, version 4.6.0). The list of differentially expressed genes was imported to the KEGG database for functional enrichment analysis. In addition, the plugin “GeneMANIA” from Cytoscape was employed to generate a protein‐interaction network.

To better understand the underlying mechanisms of therapeutic effects of Ext.R, similarities between the gene expression profile of Ext.R and chemical drugs were assessed through the CMAP database (https://portals.broadinstitute.org/cmap/). The set of genes encoding transcription factors (102 upregulated and 224 downregulated) was imported to the CMAP database to query chemical profiles. Similarity was measured by connectivity score, ranging from −1 to 1. From the “Detailed Results” of the CMAP output, chemicals with positive scores were interested, indicating that the chemicals can simulate the gene expression changes caused by Ext.R. Chemicals with high “‘Scores”’ in the “Detailed Results” were selected.

### MS‐Based Kinase Inhibitory Assay for Screening SGK1 Inhibitor

Synthesis of CKRPRAASFAE was prepared by Shanghai Sangong Biotech. In order to ensure the phosphorylation reaction of SGK1, SGK1 (0.25 µg mL^−1^) was added to kinase buffer (25 mmol Tris [pH 7.5], 5 mmol *β*‐glycerolphosphate, 2 mmol DTT, 10 mmol MgCl_2_) with the substrate peptide CKRPRAASFAE (25 µmol) and ATP (200 µmol) and incubated for 1 h at 30 °C in the presence or absence of inhibitors. A concentration‐dependent assay for SGK1 at different concentrations (0.05, 0.1, 0.2, 0.25, and 0.3 µg mL^−1^) was performed. Reactions were terminated by the addition of methyl alcohol with 0.1% trifluoroacetic acid (TFA), and supernatants were analyzed by the Agilent 1100 LC system (Agilent Technologies, USA) and a 1946D single quadrupole mass spectrometer. The acquisition parameters for LC/MS were set for positive ionization mode and selected ion monitoring (SIM) m/z values of 618.6 and 658.7 for the substrate peptide and product peptide respectively. Chromatographic separation was performed by a reversed‐phase ZORBAX SB‐C_18_ analytical column. The mobile phase included water containing 0.1% v/v TFA (A) and acetonitrile containing 0.1% v/v TFA (B). The flow rate was 0.6 mL min^−1^. A gradient program was carried out according to the following profile: 0 min, 13% B; 15 min, 28% B; 16 min, 100% B. The constituents of Ext.R were screened by the assay to evaluate their inhibitory effects on SGK1 activity. A minimum of three replicates of each sample were analyzed and averaged together. Inhibition of SGK1 was calculated by the following equation

(1) $$\mathrm{Inhibition}\;\mathrm{rate}\left(\%\right)=\left(P_{C}-P_{S}\right)/P_{C}$$

*P*_C_ and *P*_S_ represented the peak area of product peptide in the control group without test compounds and the tested sample group with various test compounds, respectively.

### Cell Lines, Cell Culture, and Transfection

The MCF‐7 cells and HEK293T cells were obtained from the cell bank of the Shanghai Institutes for Biological Sciences, and cultured in 4.5 g L^−1^ glucose Dulbecco's modified Eagle medium (DMEM, Corning) supplemented with 10% fetal bovine serum (FBS), 100 U mL^−1^ penicillin, and 100 µg mL^−1^ streptomycin. Cells were incubated at 5% CO_2_ at 37 ℃.

SGK1 mutant plasmids and adenovirus were constructed by Vigene Bioscience. All the transfection assays were performed using Lipofectamine 2000 according to the manufacturer's protocol. Adenovirus (Ad‐^S422D^SGK1) was diluted in DMEM and administered at a dose of 100 MOI in primary cardiomyocytes.

### Primary Cultures of Cardiomyocytes

Primary cultures of cardiac myocytes were prepared from neonatal rat hearts using the Neonatal Heart Dissociation Kit (MACS, Germany) according to the manufacturer's protocol. Cardiomyocyte cultures were plated in gelatin‐coated plates in a plating medium containing 10% serum. After 24 h of plating, the medium was then replaced with a serum‐free maintenance medium and incubated for another 24 h before being used for further study.

For phenylephrine (PE)‐mediated cardiac hypertrophy, cardiomyocytes cultured in 6‐well plates were stimulated with PE (50 µmol) for 48 h in serum‐free medium with or without Ext.R or HBT (50 µmol) treatment. Cardiomyocytes were lysed in cell lysis buffer to isolate protein or total RNA for western blotting or PCR analysis.

### Immunoprecipitation Assay

For the endogenous interaction assay, cardiomyocytes were treated with HBT or vehicle. After 24 h, cells were lysed with cell lysis buffer contained PMSF (Phenylmethylsulfonyl fluoride, a protease inhibitor). Protein extracts were harvested and immunoprecipitated with anti‐SGK1 or anti‐IgG antibody and protein A/G beads, and then measured by western blotting analysis.

### Docking and Binding Site Detection

The initial crystal structures of SGK1 (PDB entry: 3HDN, 3HDM, and 2R5T) were obtained from the Protein Data Bank (PDB). The prepared inhibitors were then docked by Glide with the extra precision (XP) scoring mode to estimate the binding affinity for each protein–ligand complex. The detailed process of docking has been described previously.^[^ ^46^ ^]^ HEK293T cells were transfected with SGK1 mutant plasmids. After 24 h, cells were lysed and immunoprecipitated with anti‐FLAG antibody and beads. Immune complexes were washed with kinase buffer and incubated in kinase buffer with substrate peptide for 1 h at 30 ℃ followed by LC/MS measurement as described above. KINOMEscan Profiling Service and KinaseProfiler Service Assay were provided by Eurofins Discovery.

### Statistical Analysis

The data were interpreted as means ± SD of three or more independent experiments. Statistical analysis with unpaired two‐tailed t test for comparisons between two groups and one‐way analysis of variance (ANOVA) and Dunnett correction for comparisons among more than two groups were carried out using GraphPad Prism (GraphPad Software, SanDiego, CA). *P*‐values less than 0.05 were considered statistically significant.

## Conflict of Interest

The authors declare no conflict of interest.

## Supporting information

Supporting Information

Supplemental Table 1

## Data Availability

The data that support the findings of this study are available from the corresponding author upon reasonable request.
