# Supplementary material for: Discovery of Herbacetin as a Novel SGK1 Inhibitor to Alleviate Myocardial Hypertrophy
Source: Adv Sci (Weinh). 2021 Nov 10;9(2):2101485. doi: 10.1002/advs.202101485 (PMC8805583; doi:10.1002/advs.202101485)
Supplement: Supplementary file 1 — Supporting Information [file ADVS-9-2101485-s001.pdf]

© 2021 Wiley-VCH GmbH

ADVANCED SCIENCE
Open Access

Supporting Information

for *Adv. Sci.,* DOI: 10.1002/advs.202101485

Discovery of Herbacetin as a novel SGK1 inhibitor to alleviate myocardial hypertrophy

Shujing Zhang, Yingchao Wang, Min Yu, Ye Shang, Yanxu Chang, Hong Zhao, Yu Kang, Lu Zhao, Lei Xu, Xiaoping Zhao, Dario Difrancesco, Mirko Baruscotti, Yi Wang**

Supporting Information

Discovery of Herbacetin as a novel SGK1 inhibitor to alleviate myocardial hypertrophy

Shujing Zhang, Yingchao Wang, Min Yu, Ye Shang, Yanxu Chang, Hong Zhao, Yu Kang, Lu Zhao, Lei Xu, Xiaoping Zhao, Dario Difrancesco, Mirko Baruscotti, Yi Wang**

Table of Contents

I. Supplementary results

Figure S1. Effect of Ext.R on Sham operated Mice.

Figure S2. Serum biochemical parameters of mice in Sham and Sham + Ext.R groups.

Table S1. List of DEGs regulated by Ext.R

Table S2. The correlated compounds in Connectivity Map analysis.

Figure S3. Key nodes of FoxO signaling pathway regulated by Ext.R. generated by IPA analysis.

Figure S4. The phosphorylation level of FoxO1 and GSK3β in myocardial tissues suffering from pressure overload.

Figure S5. Mass spectrometry-based kinase inhibitor assay for SGK1 inhibitors screening.

Figure S6. IC_{50} value of structurally related flavonoids against SGK1.

Table S3. Characterization of chemical constituents in Ext.R by UPLC-Q-TOF-MS.

Table S4. Compounds docking scores for SGK1 PDB models.

Figure S7. Screening active compounds of anti-hypertrophy from Ext.R.

Figure S8. Western blot analysis of related proteins in PE-treated cardiomyocytes.

Figure S9. Western blot analysis of NF-κB p65, Nedd4l and NDRG1.

Figure S10. Immunofluorescent analysis of the expression of α-smooth muscle actin in cardiomyocytes.

Figure S11. HBT blocked ISO-induced FoxO1 phosphorylation, oxidative stress, and calcium accumulation in vivo.

Figure S12. Effect of HBT on control mice.

Figure S13. Serum level of biochemical markers in mice of Ctrl and Ctrl + HBT groups.

Figure S14. Plasma concentration (A) and *in vivo* distribution (B) of HBT after single oral

administration.

Figure S15. Murcko scaffolds and Molecular fingerprint similarity.

Table S5. Molecular Docking of 9 SGK1 inhibitors and HBT.

Figure S16. UPLC-Q-TOF-MS base peak intensity chromatograms of *Rhodiola* species.

II. Supplementary methods

1. Reagents
2. Phalloidin Staining and Immunofluorescence
3. ROS and Ca^{2+} detection
4. Histology and Immunohistochemistry (IHC)
5. RT-PCR of mRNA levels
6. Pharmacokinetics studies
7. Western blot
8. Statistical Analysis

III. Supplementary tables for methods

Table S6. Sequences of primers used for RT-PCR analysis and plasmid constructions

I. Supplementary results

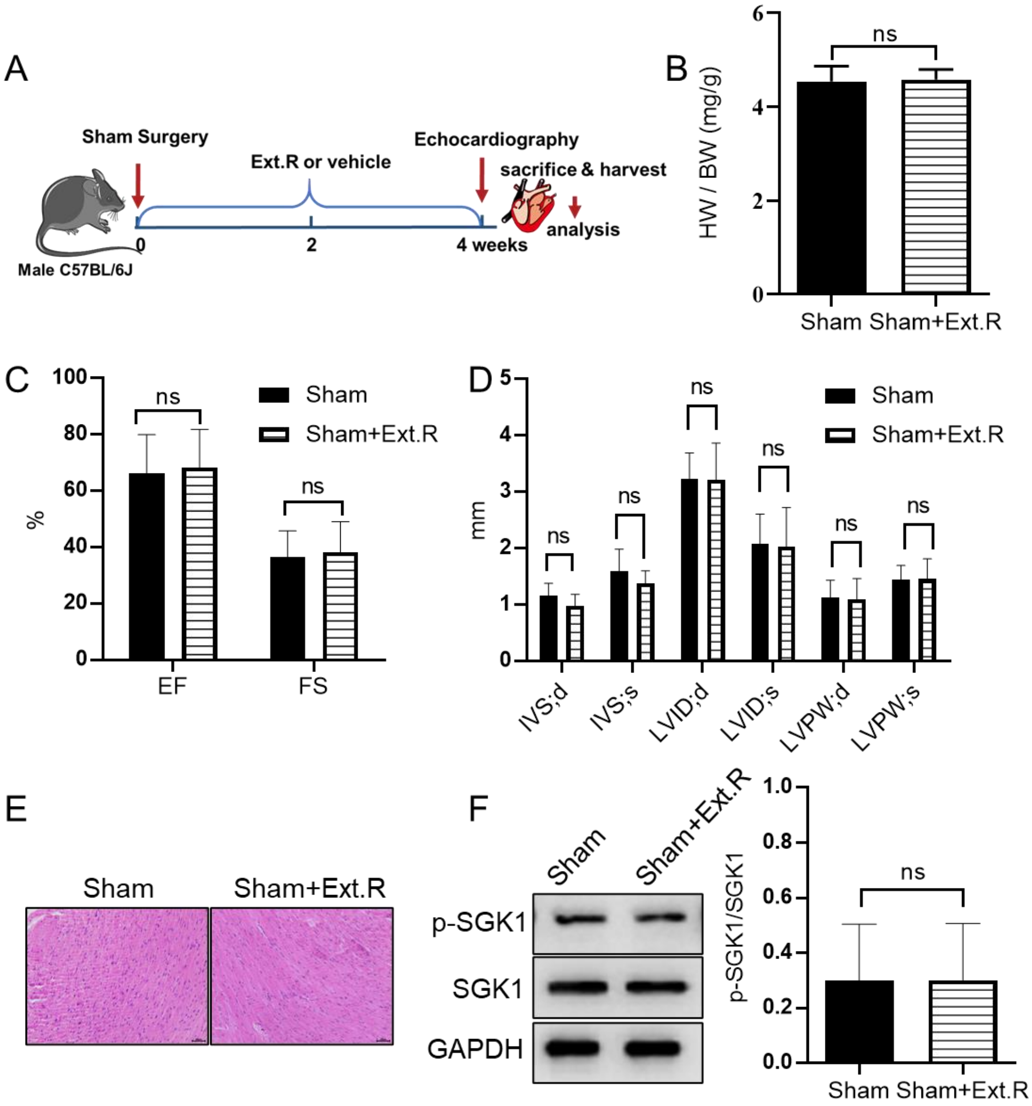

Figure S1. The mice were divided into two groups: Sham with saline, Sham mice treated with Ext.R. (A) Schedule of animal treatments. (B) Heart weight/Body weight ratio (HW/BW) was detected in Sham and Sham + Ext.R group. (C-D) Echocardiography assessments were performed in the two groups. n = 9-12. (E) Hematoxylin-eosin staining（HE）in different groups of mice. n = 4, Scale bar=50μm. (F) The phosphorylation level of SGK1 in myocardial tissues in the two groups (Sham, Sham + Ext.R), n = 4. Data were analyzed using unpaired two-tailed t test and data were expressed as means ± SD, ns: no significant.

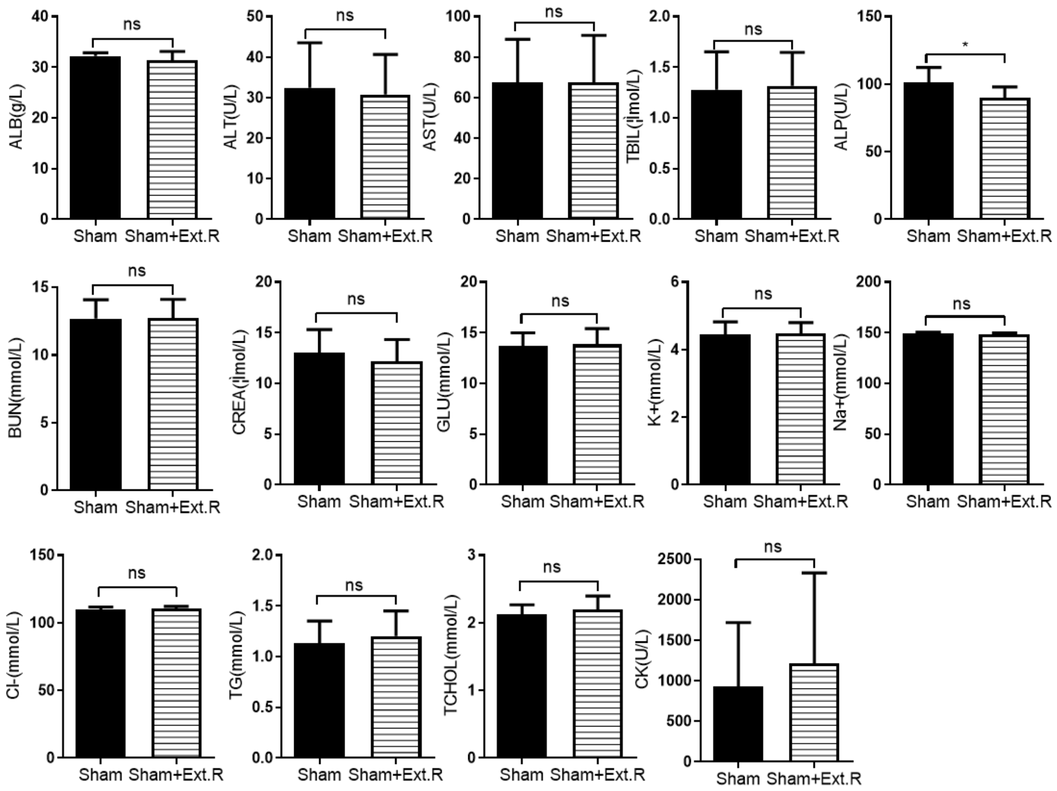

Figure S2. Serum biochemical parameters of mice in Sham and Sham + Ext.R groups. Data were analyzed using unpaired two-tailed t test and data were expressed as means ± SD, ns: no significant.

Table S2. The correlated compounds in Connectivity Map analysis

Rank	CMap name	Dose	Cell	Score
1	phenoxybenzamine	12 μM	MCF7	1
2	quercetin	12 μM	MCF7	0.987
3	primaquine	9 μM	MCF7	0.981
4	syrosingopine	6 μM	MCF7	0.974
5	15-delta prostaglandin J2	10 μM	MCF7	0.936
6	rottlerin	10 μM	MCF7	0.934
7	equilin	15 μM	MCF7	0.925
8	5253409	17 μM	MCF7	0.922
9	MG-132	21 μM	MCF7	0.901
10	thiostrepton	2 μM	MCF7	0.896
11	lomustine	100 μM	MCF7	0.892
12	5182598	25 μM	MCF7	0.878
13	mebendazole	14 μM	MCF7	0.874
14	lynestrenol	14 μM	MCF7	0.872
15	5224221	12 μM	MCF7	0.872
16	terfenadine	8 μM	MCF7	0.863
17	withaferin A	1 μM	MCF7	0.862
18	ionomycin	2 μM	MCF7	0.854
19	F0447-0125	10 μM	MCF7	0.853
20	thapsigargin	100 nM	MCF7	0.853
21	5114445	10 μM	MCF7	0.844
22	tyrphostin AG-1478	32 μM	MCF7	0.843
23	5155877	10 μM	MCF7	0.842
24	tribenoside	8 μM	MCF7	0.838
25	STOCK1N-35696	15 μM	MCF7	0.837
26	semustine	100 μM	MCF7	0.832
27	hexetidine	12 μM	MCF7	0.826
28	parthenolide	16 μM	MCF7	0.825
29	5109870	25 μM	MCF7	0.82
30	securinine	18 μM	MCF7	0.816
31	nocodazole	13 μM	MCF7	0.812
32	valinomycin	100 nM	MCF7	0.811
33	kaempferol	14 μM	MCF7	0.809
34	prasterone	12 μM	MCF7	0.809
35	prenylamine	10 μM	MCF7	0.806
36	suloctidil	12 μM	MCF7	0.806
37	0179445-0000	10 μM	MCF7	0.806

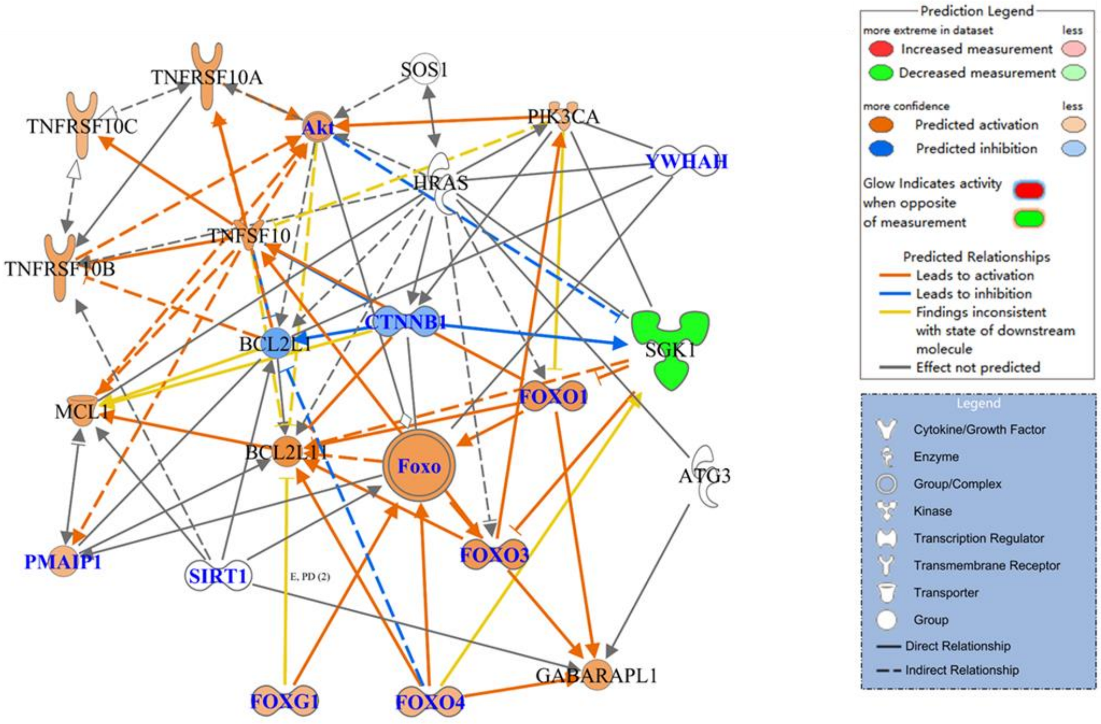

Figure S3. Key nodes of FoxO signaling pathway regulated by Ext.R. generated by IPA analysis.

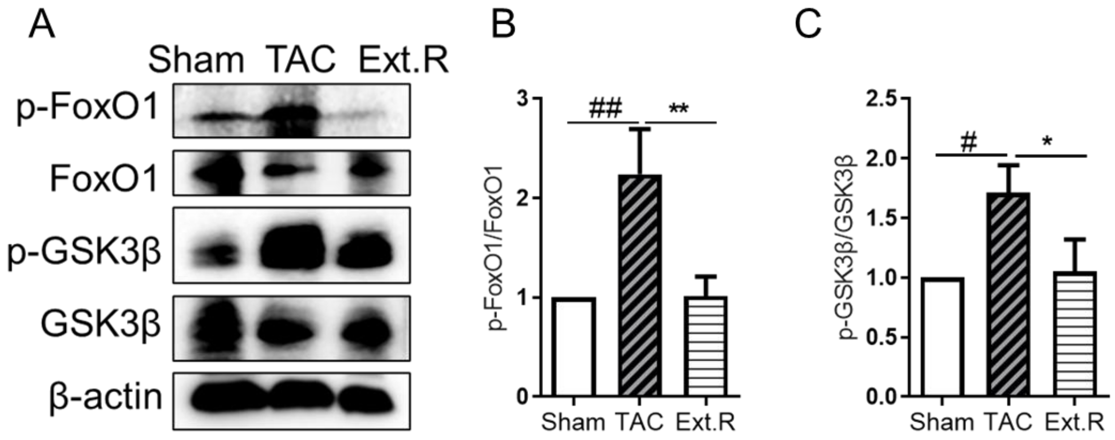

Figure S4. The phosphorylation level of FoxO1 and GSK3β in myocardial tissues suffering from pressure overload, n=3 for each group, data were analyzed using one-way ANOVA and data were expressed as means ± SD, ##p<0.01 versus Sham group, **p<0.01 versus TAC group.

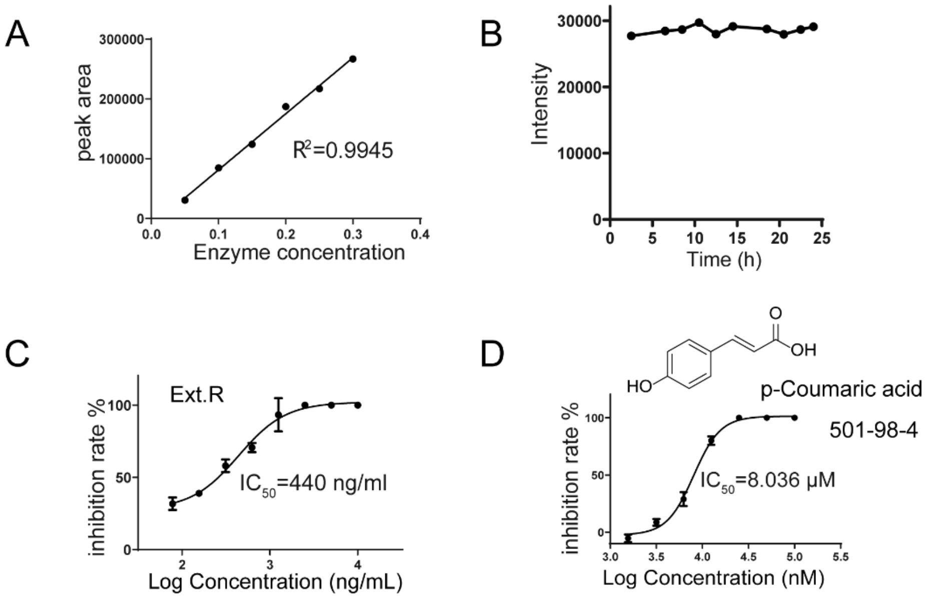

Figure S5. Mass spectrometry-based kinase inhibitor assay for SGK1 inhibitors screening. (A-B) The linear range of SGK1 concertation measured by MS-based assay (A) and stability of assay measured in 24 h (B). (C-D) Structure formula and IC_{50} value of Ext.R and p-coumaric acid to SGK1 activity.

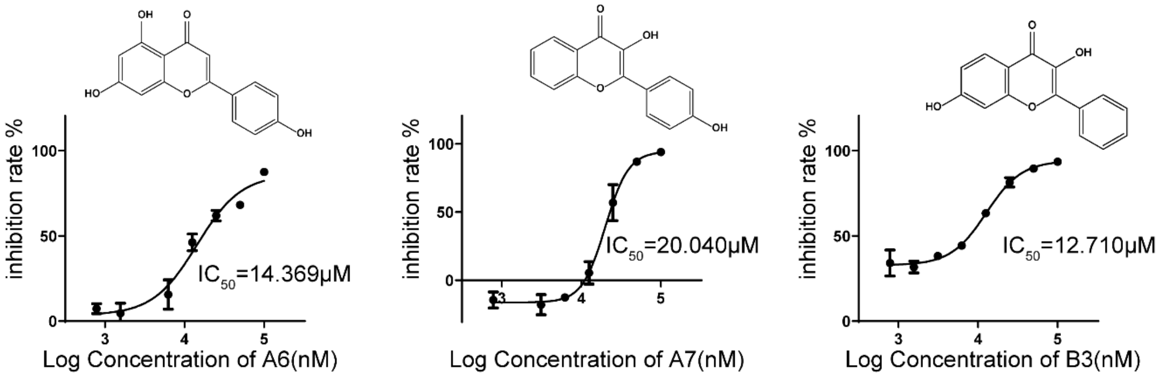

Figure S6. IC_{50} value of structurally related flavonoids against SGK1.

Table S3. Characterization of chemical constituents in Ext.R by UPLC-Q-TOF-MS.

No.	Retention time (min)	Identification	Detected ion (m/z)	Formula
1	3.038	p-Hydroxyphenacyl-β-D-glucopyranoside	313.0927[M-H]-	$C_{14}H_{18}O_8$
2	3.709	Crenulatin	293.1245[M+FA-H]-	$C_{11}H_{20}O_6$
3	3.81	*p*-Tyrosol	137.0263[M-H]-	$C_8H_{10}O_2$
4	3.946	n-Pentenyl (β-D-galactopyranosyl) -(1<*>4)-β-D-glucopyranoside	455.1759[M+FA-H]-	$C_{17}H_{30}O_{11}$
5	3.946	Salidroside	345.1186[M+FA-H]-	$C_{14}H_{20}O_7$
6	4.934	3-methyl-2-but-2-en-1-yl 6-O-α-L -arabinopyranosyl-β-D-glucopyranoside	425.1655[M+FA-H]-	$C_{16}H_{28}O_{10}$
7	5.111	Rosiridin	350.2179[M+NH$_4$]+	$C_{16}H_{28}O_7$
8	5.126	Creoside I	349.1503[M+FA-H]-	$C_{14}H_{24}O_7$
9	5.188	Rhodioloside D	395.1915[M+FA-H]-	$C_{16}H_{30}O_8$
10	5.239	p-Hydroxy benzaldehyde	121.0318[M-H]-	$C_7H_6O_2$
11	5.56	Viridoside	313.1287[M-H]-	$C_{15}H_{22}O_7$
12	5.639	Caffeic acid	179.0361[M-H]-	$C_9H_8O_4$
13	6	Creoside II or its isomer	351.1656[M+FA-H]-	$C_{14}H_{26}O_7$
14	6.299	Procyanidin B3	577.1347[M-H]-	$C_{30}H_{26}O_{12}$
15	6.444	1,2,6-tri-O-galloyl β-D-glucopyranoside	635.0885[M-H]-	$C_{27}H_{24}O_{18}$
16	6.818	Catechin	289.0716[M-H]-	$C_{15}H_{14}O_6$
17	7.202	Ethyl gallate	197.0457[M-H]-	$C_9H_{10}O_5$
18	7.356	2-phenylethyl 1-O-α-L-arabinopyranosyl-(1->6)-β-D-glucopyranoside	435.2069[M+NH$_4$]+	$C_{19}H_{28}O_{10}$
19a	7.357	2-(4-hydroxyphenyl)ethyl β-D-glucopyranosyl-	461.1652[M-H]-	$C_{20}H_{30}O_{12}$

		(1→6)-β-D-glucopyranoside		
20	7.462	p-Coumaric acid	163.0407[M-H]-	$C_9H_8O_3$
21	8.039	6-O-galloylsalidroside	451.1232[M-H]-	$C_{21}H_{24}O_{11}$
22	8.417	Rutin/Kaempferol-3-O-sophoroside	609.1458[M-H]-	$C_{27}H_{30}O_{16}$
23	8.683	8-hydroxykaempferol 3-O-β-glucopyranosyl -7-O-β-D-glucopyranosyl-(1->3)-α-L-rhamnopyranoside	773.2156[M+H]+	$C_{33}H_{40}O_{21}$
24	8.947	5'-methoxy-8'-hydroxy-(+)-isolariciresinol-4'-O-β-D-glucopyranoside	567.2073[M-H]-	$C_{27}H_{36}O_{13}$
25	9.254	Rhodioloside E	511.2386[M+FA-H]-	$C_{21}H_{38}O_{11}$
26	9.674	1,2,3,6-tetra-O-galloyl-β-D-glucose	787.1004[M-H]-	$C_{34}H_{28}O_{22}$
27	10.063	Epicatechin gallate	441.0816[M-H]-	$C_{22}H_{18}O_{10}$
28	10.621	Rosarin	473.1649[M+FA-H]-	$C_{20}H_{28}O_{10}$
29	10.725	Isoquercetin	465.1035[M+H]+	$C_{21}H_{20}O_{12}$
30	10.773	Foliasalacioside B1	549.2546[M+FA-H]-	$C_{24}H_{40}O_{11}$
31	12.449	(3R)-octa-1-en-3-yl O-[β-D-xylopyranosyl -(1''→6')]-β-D-glucopyranoside	467.2118[M+FA-H]-	$C_{19}H_{34}O_{10}$
32	13.391	Sacranoside A/sachaloside II	491.2124[M+FA-H]-	$C_{21}H_{34}O_{10}$
33	13.642	Epicatechin	289.0708[M-H]-	$C_{15}H_{14}O_6$
34	13.897	(-)-Epicatechin-4β->8-(-)-epicatechin-3-O-gallate	729.1473[M-H]-	$C_{37}H_{30}O_{16}$
35	14.273	Crenuloside or isomer	593.1508[M-H]-	$C_{27}H_{30}O_{15}$
36a	14.616	Kaempferol-7-O-α-L-rhamnoside	431.0967[M-H]-	$C_{21}H_{20}O_{10}$
37a	15.482	Rhodiosin	609.146[M-H]-	$C_{27}H_{30}O_{16}$
38	15.82	Herbacetin	301.0341[M-H]-	$C_{15}H_{10}O_7$
39	15.82	Rhodionin	447.0916[M-H]-	$C_{21}H_{20}O_{11}$

40	16.795	Crenuloside or kaempferol-3-rutinoside	595.1671[M+H]+	$C_{27}H_{30}O_{15}$
41	17.096	Quercetin	301.0341[M-H]-	$C_{15}H_{10}O_7$
42	17.129	Rhodioloside C	493.2275[M-H]-	$C_{22}H_{38}O_{12}$
43	17.377	Rhodiooctanoside	469.2271[M+FA-H]-	$C_{19}H_{36}O_{10}$
44	17.582	Kenposide A	493.2277[M+FA-H]-	$C_{21}H_{36}O_{10}$
45	17.893	Luteolin 7-O-α-L-Rhamnoside	431.0972[M-H]-	$C_{21}H_{20}O_{10}$
46	19.98	Creoside V	495.2437[M+FA-H]-	$C_{21}H_{38}O_{10}$
47	21.42	Luteolin	285.0394[M-H]-	$C_{15}H_{10}O_6$
48	27.617	12β-Benzoyloxy-20-oxo-17α-H-pregnen-(5)-diol-(3β,14β); 'Benzoylramanon'	497.2591[M+FA-H]-	$C_{28}H_{36}O_5$

Table S4. Compounds docking scores for SGK1 PDB models.

Compounds	SGK1 PDB models Glide score		
	2R5T	3HDN	3HDM
Herbacetin	-10.759	-10.338	-10.026
Rhodiosin	-9.838	-10.422	-11.389
p-coumaric acid	-10.436	-6.221	-5.77
EMD638683	-7.742	-10.721	-10.564

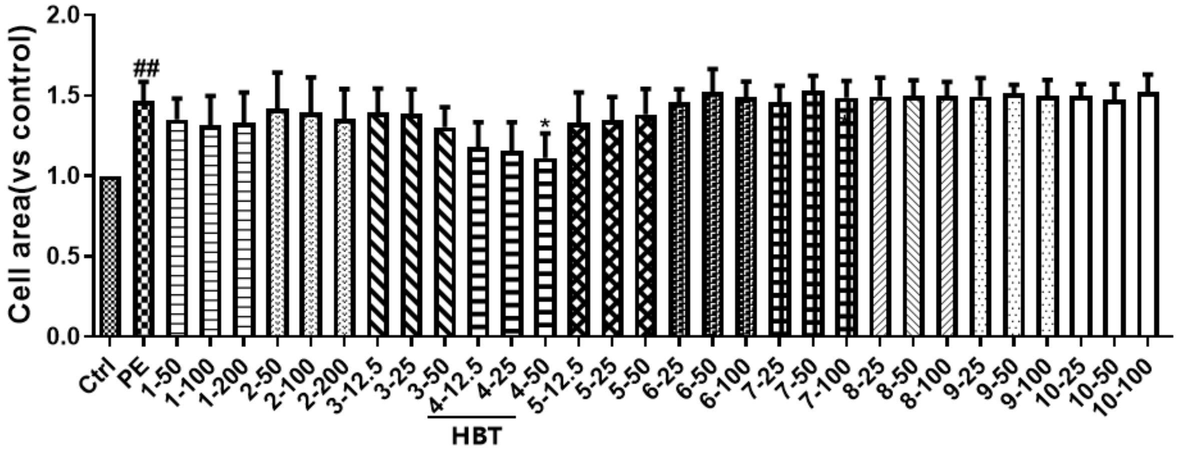

Figure S7. Screening active compounds of anti-hypertrophy from Ext.R. The number of 1-10 represent Salidroside, Tyrosol, Rhodiosin, Herbacetin, Rhodionin, Rosiridin, Rosavin ,Rosarin, p-coumaric acid and syringic acid. 4-12.5 ,4-25 ,4-50 respectively represent HBT (12.5 μmol), HBT(25 μmol) and HBT(50 μmol). Data were analyzed using one-way ANOVA and data were expressed as means ± SD, ##*p*<0.01 versus Ctrl group, **p*<0.05 versus PE group.

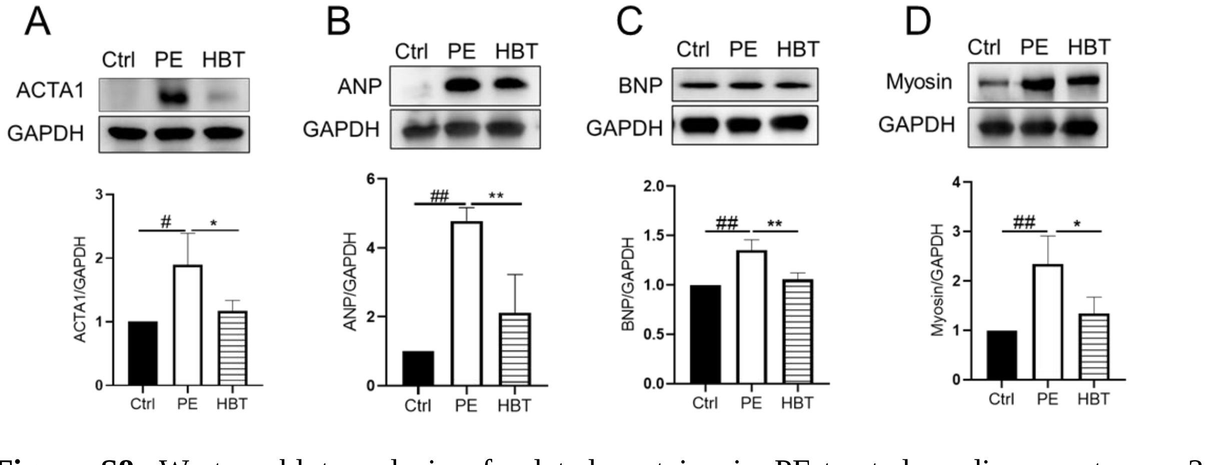

Figure S8. Western blot analysis of related proteins in PE-treated cardiomyocytes, n=3 independent experiments. Data were analyzed using one-way ANOVA and data were

expressed as means ± SD, ##*p*<0.01 versus Ctrl group, #*p*<0.05 versus Ctrl group, ***p*<0.01 versus PE group, **p*<0.05 versus PE group.

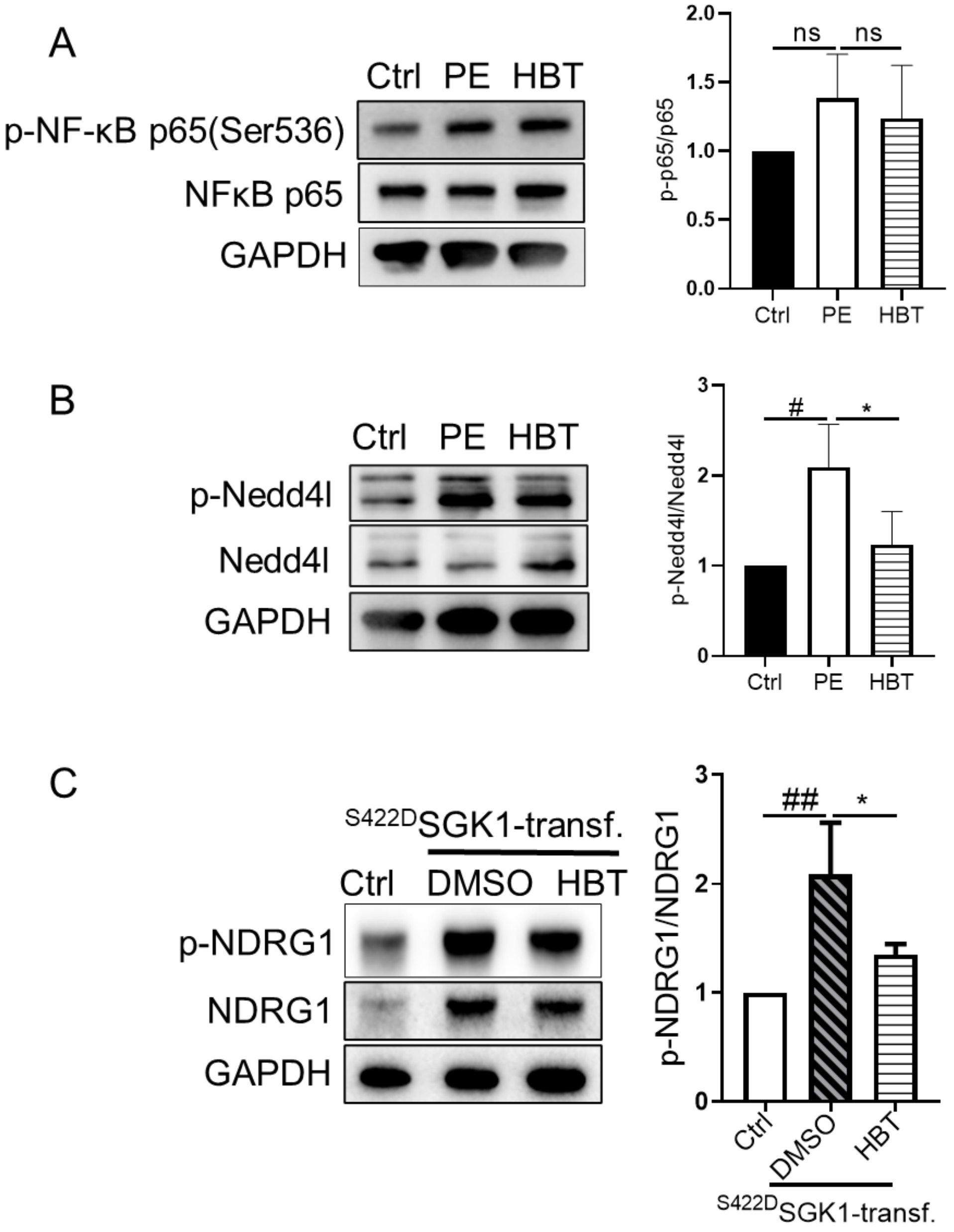

Figure S9. (A-B) Western blot analysis of related proteins (NF-κB p65 and Nedd4l) in PE-treated cardiomyocytes (n=3). All data were analyzed using one-way ANOVA and data are expressed as means ± SD, #p<0.05 versus Ctrl group, *p<0.05 versus PE group, ns: no significant. (C) Western blot analysis of NDRG1 in non-transfected (control adenovirus) and in ^{S422D}SGK1-transfected primary cardiac myocytes treated with DMSO as solvent control (^{S422D}SGK1-transf. group) or HBT (50μmol, ^{S422D}SGK1-transf.+HBT group), n=3 independent experiments. All data were analyzed using one-way ANOVA and data were

expressed as means ± SD, ##p<0.01 *versus* Ctrl group, *p<0.05 *versus* ^{S422D}SGK1-transf. group (DMSO as solvent control).

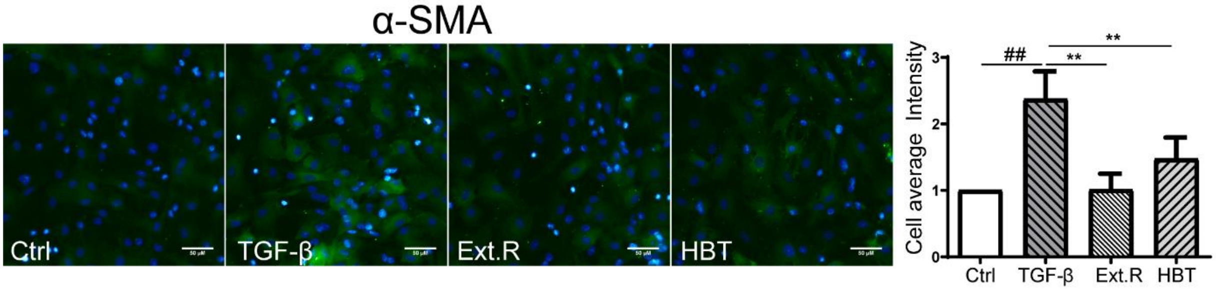

Figure S10. Immunofluorescent analysis of the expression of α-smooth muscle actin in cardiomyocytes. Cardiomyocytes were treated with 10 ng/ml transforming growth factor (TGF)-β1 for 48 h. Green represents α-SMA and blue represents nuclei. Data were analyzed using one-way ANOVA and data were expressed as means ± SD, ##p<0.01 versus Ctrl group, **p<0.01 versus TGF-β group.

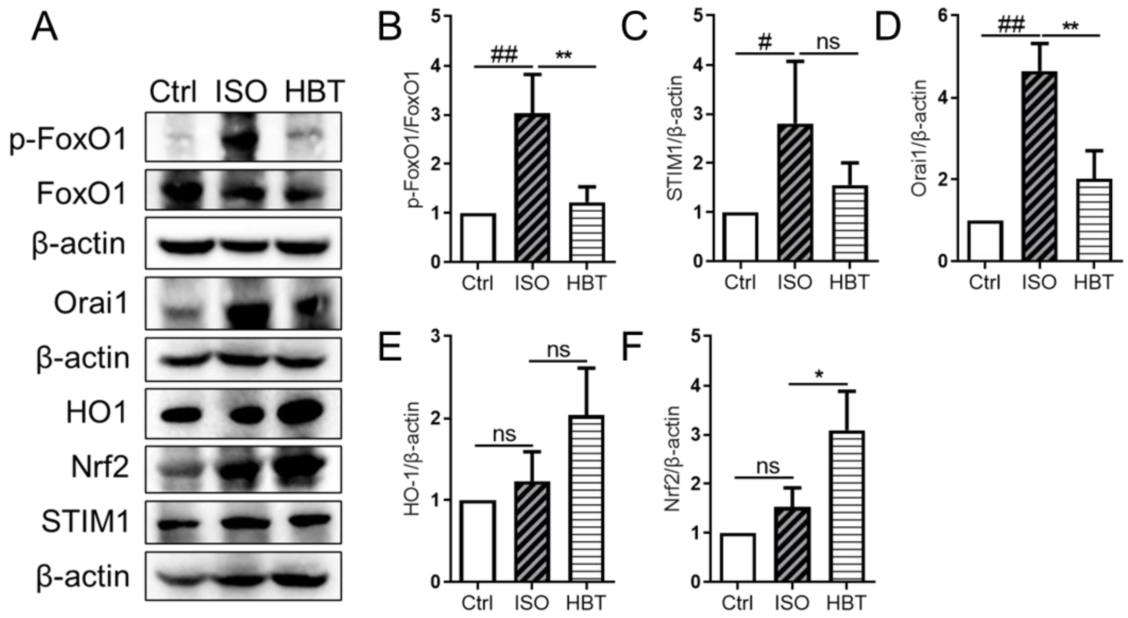

Figure S11. HBT blocked ISO-induced FoxO1 phosphorylation, oxidative stress, and calcium accumulation in vivo. Data were analyzed using one-way ANOVA and data were expressed as means ± SD, ##p<0.01 versus Ctrl group, **p<0.01 versus ISO group, *p<0.05 versus ISO group, ns: no significant.

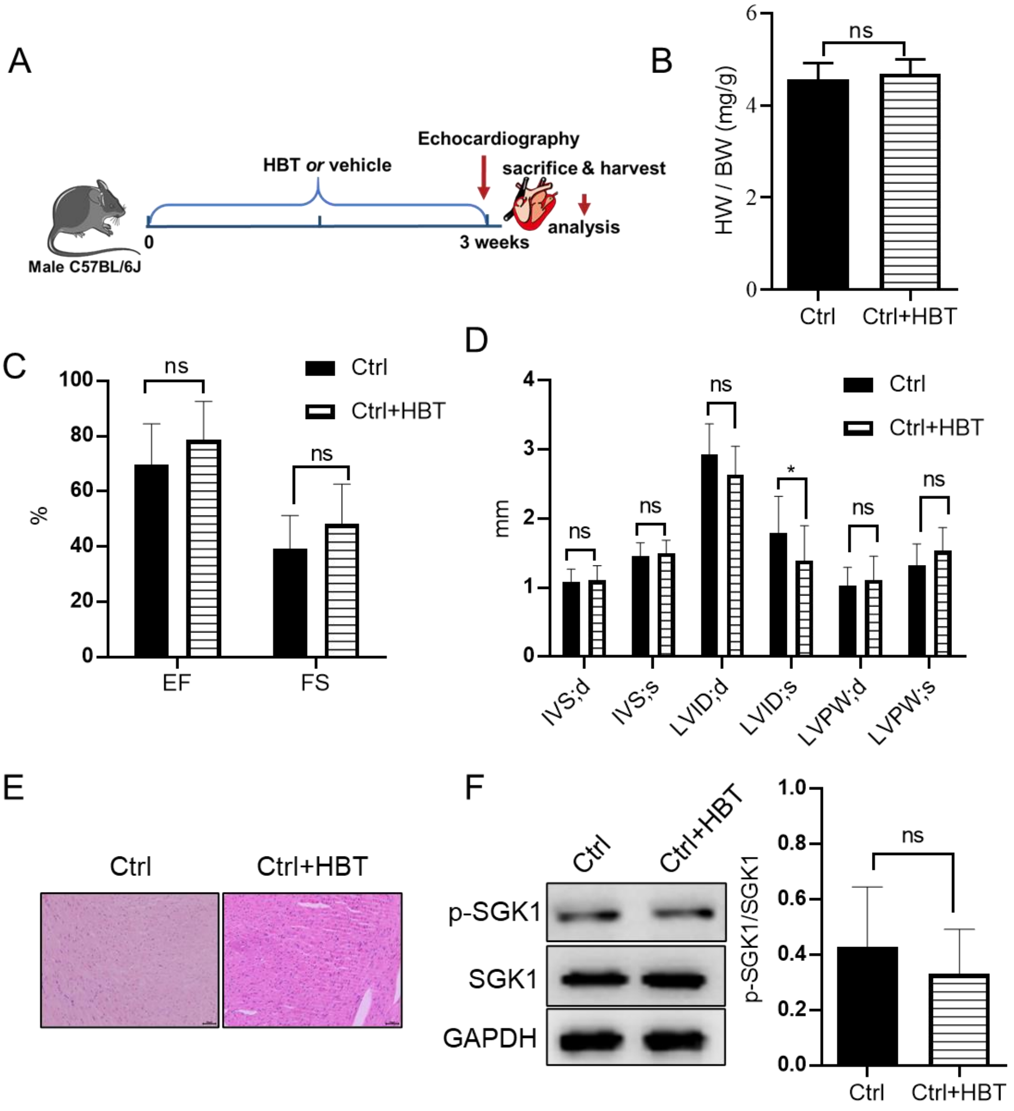

Figure S12. The mice were divided into two groups: Sham, Sham mice treated with HBT (Ctrl + HBT). (A) Schedule of animal treatments. (B) Heart weight/Body weight ratio (HW/BW) was detected in Ctrl and Ctrl + HBT group. (C-D) Echocardiography assessments were performed in the two groups. n=10-12. (E) HE in different groups of mice. n=4, Scale bar=50 μm. (F) The phosphorylation level of SGK1 in myocardial tissues in the two groups (Ctrl, Ctrl + HBT), n=4. Data were analyzed using unpaired two-tailed t test and data were expressed as means ± SD, ns: no significant.

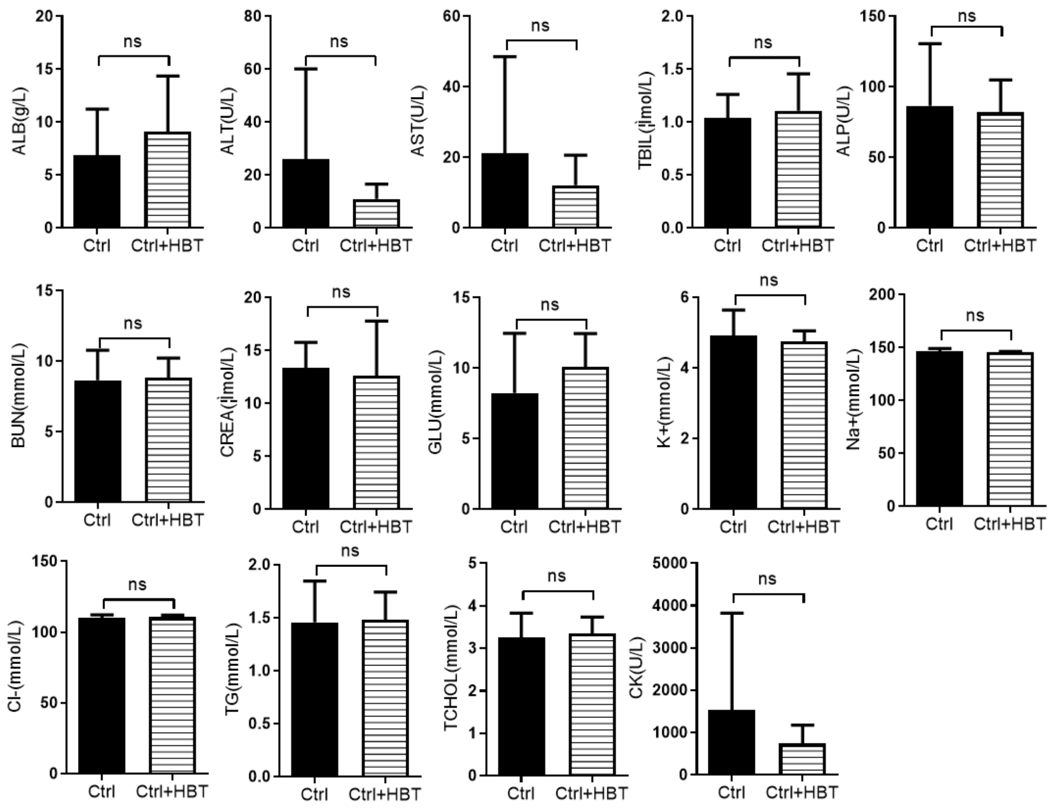

Figure S13. Serum level of biochemical markers in mice of Ctrl and Ctrl + HBT groups. Data were analyzed using unpaired two-tailed t test and data were expressed as means ± SD, ns: no significant.

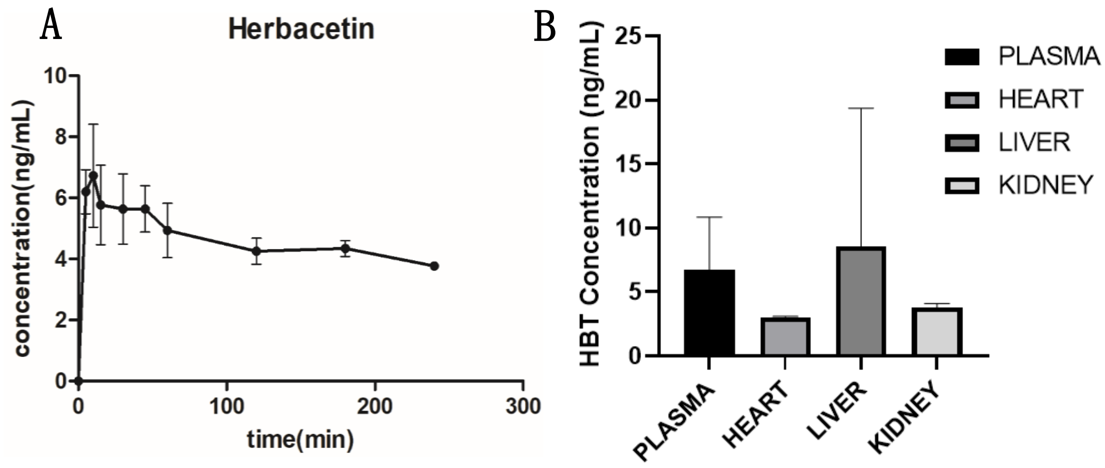

Figure S14. Plasma concentration (A) and *in vivo* distribution (B) of HBT after single oral administration with dosage of 40mg/kg rat weight (n=5-6). The quantitation of HBT was performed by LC-MS/MS analysis.

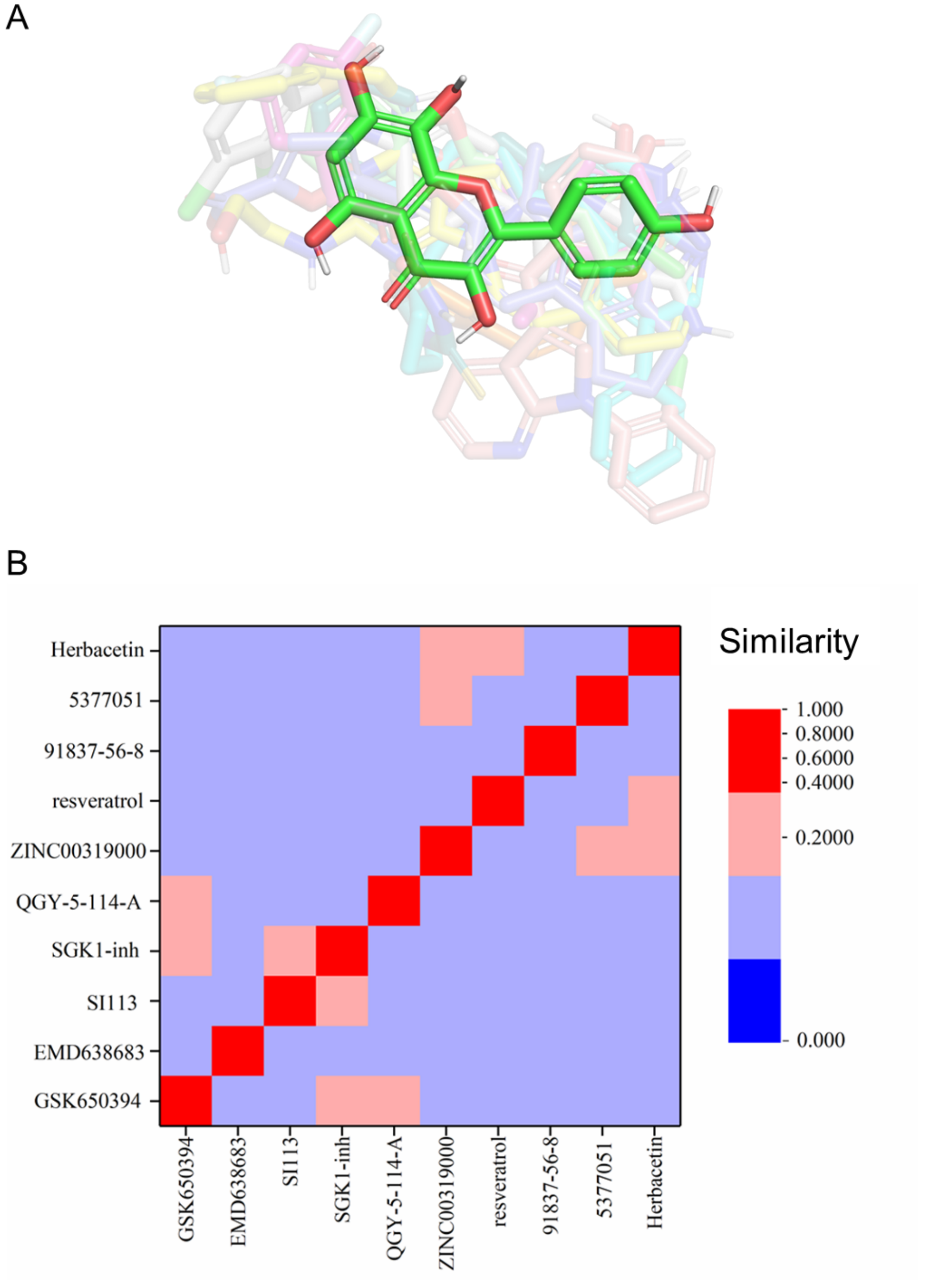

Figure S15. (A) Murcko scaffolds. (B) Molecular fingerprint similarity.

Table S5. Molecular Docking of 9 SGK1 inhibitors and HBT

Name	Predominant interactions	Key residues
GSK650394	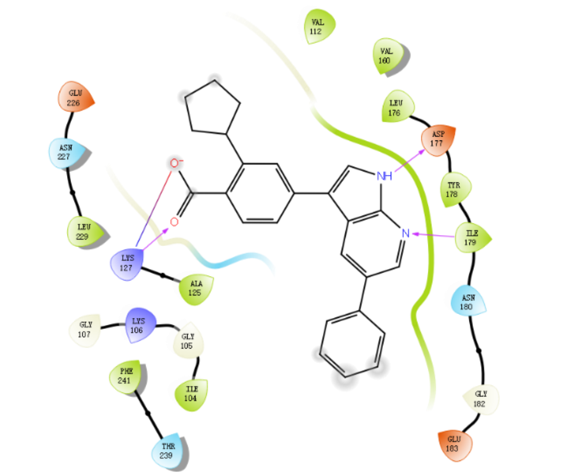	LYS127, ASP177, ILE179
EMD638683	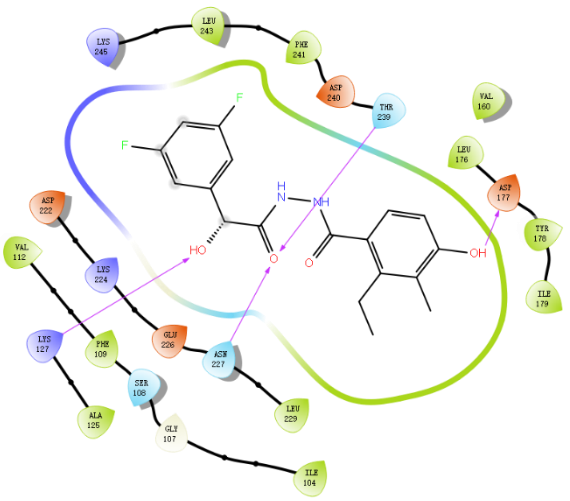	LYS127, ASN227, THR239
SI113	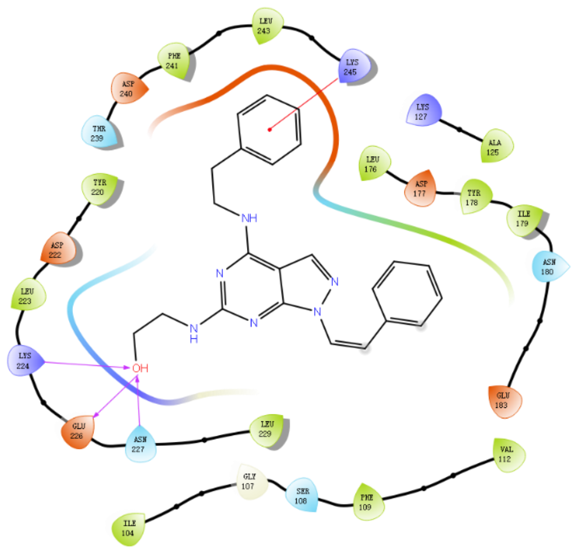	LYS224, GLU226, ASN227, LYS245

SGK1-inh	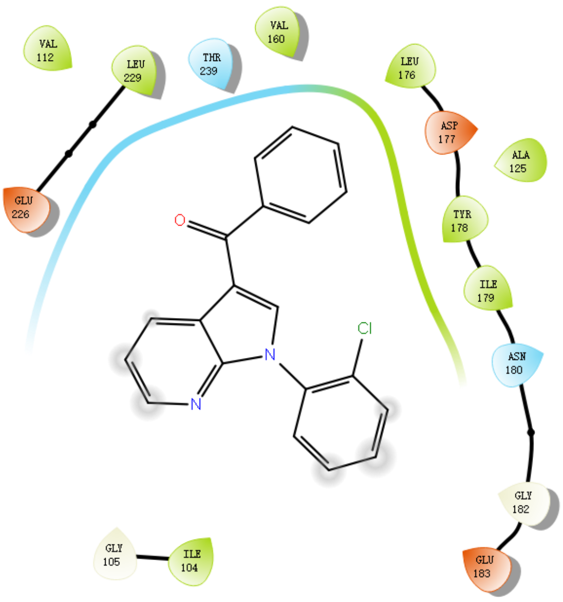	None
QGY-5-114-A	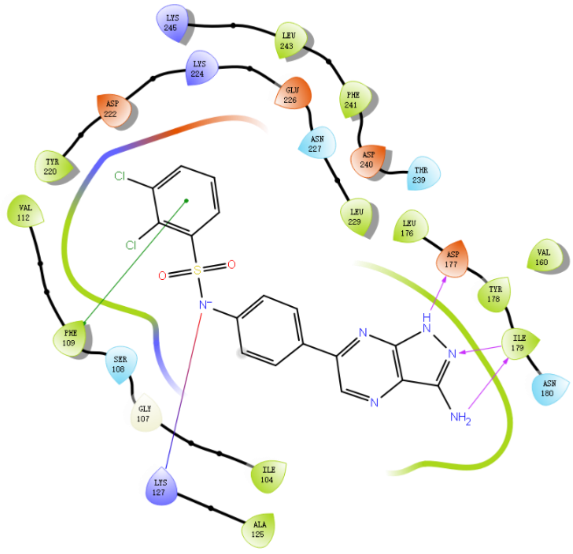	PHE109, LYS127, ASP177, ILE179

ZINC00319000	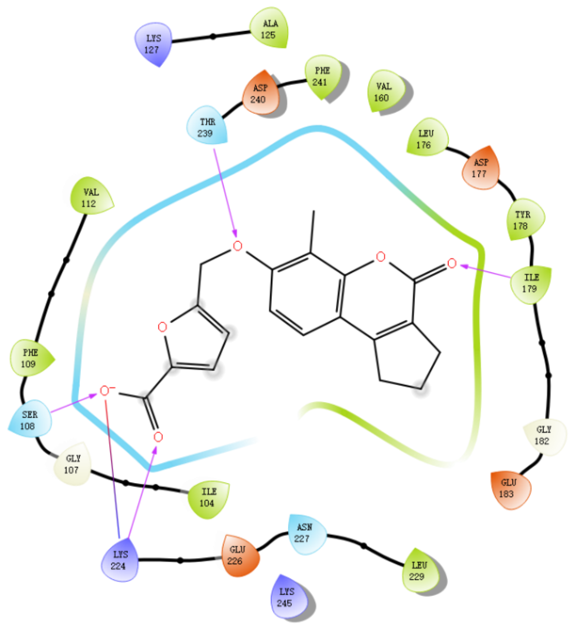	SER108, ILE179, LYS224, THR239
Resveratrol	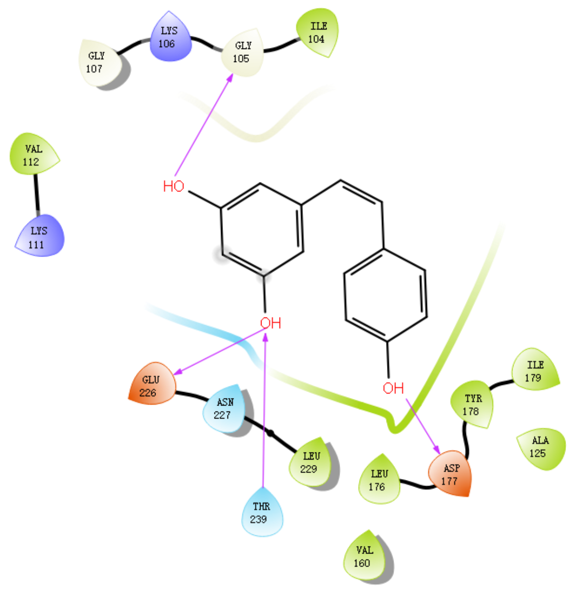	GLY105, ASP177, GLU226, THR239
1H-indole-2,3-dione 3-(1,3-benzoxazol-2-ylhydrazone)	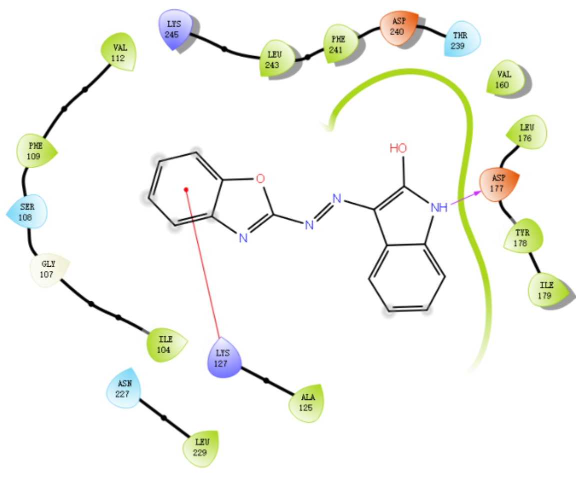	LYS127, ASP177

5377051	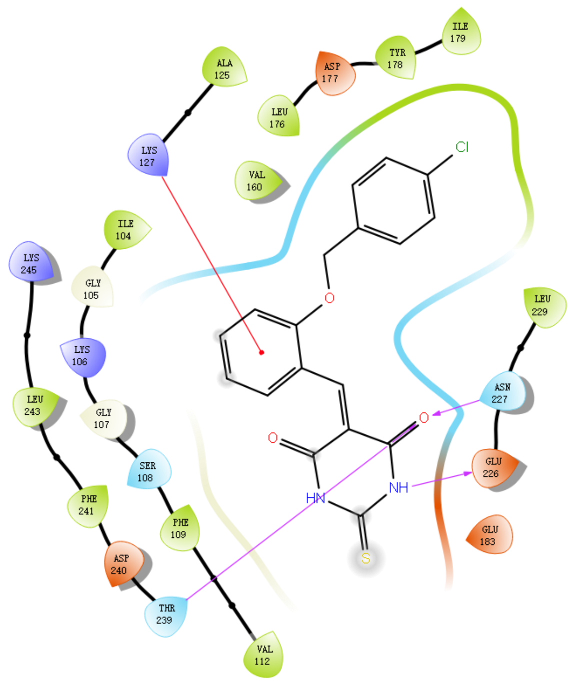	LYS127, GLU226, ASN227, THR239
Herbacetin	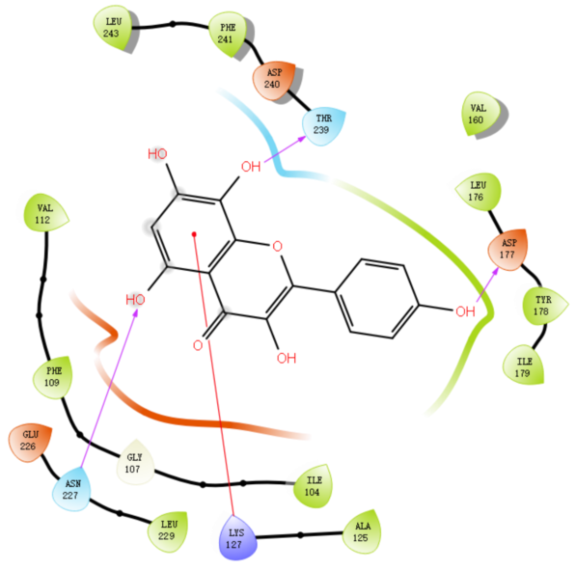	LYS127, ASP177, ASN227, THR239

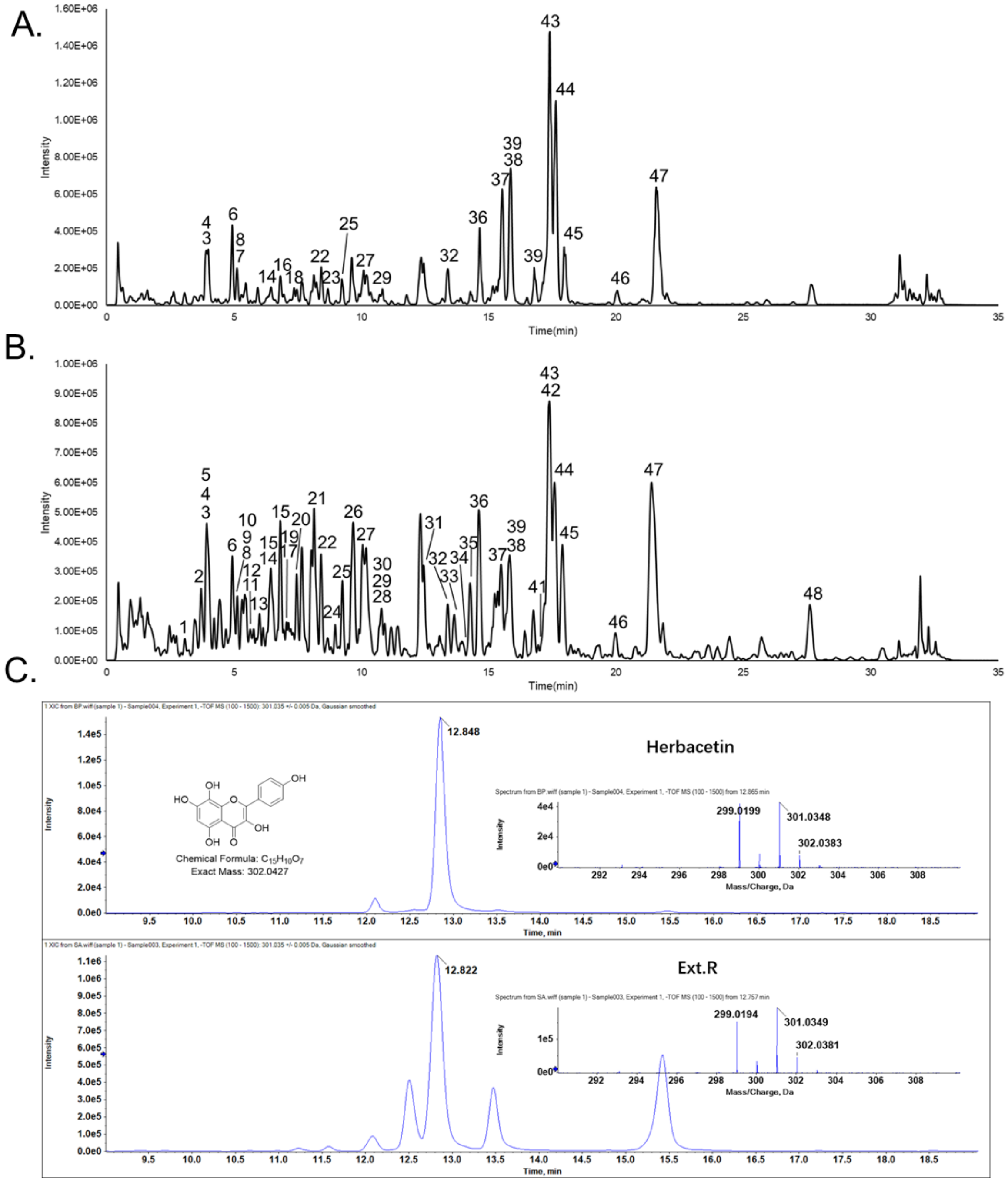

Figure S16. UPLC-Q-TOF-MS base peak intensity chromatograms of *Rhodiola* species. Total ion chromatogram positive mode (A); total ion chromatogram- negative mode (B), Herbacetin (C).

II. Supplementary methods

1. Reagents

Recombinant human SGK1 protein (ab60883) was obtained from Abcam. DCFH-DA and

Fluo-4 AM were obtained from Beyotime. SGK1 antibody and GAPDH antibody were obtained from Beyotime. FoxO1, Phospho-FoxO1 (Ser256), p44/42 MAPK (Erk1/2), Phospho-p44/42 MAPK (Erk1/2) (Thr202/Tyr204), Phospho-NF-κB p65 (Ser536), NF-κB p65, Phospho-GSK-3β (Ser9), GSK-3β, Phospho-NDRG1 (Thr346), NDRG1, Phospho-NEDD4L (Ser342), NEDD4L, HO-1, Nrf2 and MnSOD antibody were obtained from Cell Signaling. Phospho-SGK1(S422) antibody was obtained from Affinity. Orai1, STIM1, ACTA1 and FLAG antibody were obtained from Proteintech. BNP antibody was obtained from Abcam. ANP antibody was obtained from Santa Cruz Biotechnology. Myosin antibody was obtained from Sigma-Aldrich. Reference compounds were obtained from Shanghai Yuanye Biotechnology. Lipofectamine 2000 transfection reagent was purchased from Invitrogen. Protein A/G agarose was purchased from Beaver Biotechnology. Phenylephrine (PE) was obtained from Aladdin. Deionized water was prepared with an Elga PURELAB flex system (ELGA LabWater, UK).

2. Phalloidin Staining and Immunofluorescence

To determine phenylephrine-induced hypertrophy, Alexa Fluor 488 Phalloidin (Cell Signaling) was used to fluorescently stain the cytoskeleton by the binding of phalloidin to F-actin. In brief, cells were fixed for 15 min using 4% paraformaldehyde then rinsed three times in PBS. Following incubation of Alexa Fluor 488 Phalloidin and Hoechst for 15 min at room temperature, cells were rinsed once with PBS. Images were carried out using an ImageXpress Micro® Confocal High-Content Imaging System (Molecular Devices).

For immunocytochemistry, cells were fixed with 4% paraformaldehyde for 15 min and permeabilized with 0.1% Triton-X for 10 min at room temperature. After blocking with 5% BSA for 1 h, the cells were incubated overnight at 4 °C with rabbit anti-FoxO1 antibody and then with goat anti-rabbit AlexaFluor 488–conjugated antibody for 1 h at room temperature. Nuclei were stained with Hoechst. Images were acquired using a high-resolution laser confocal microscope (Leica) using an oil immersion A-Plan ×63 objective.

3. ROS and Ca^{2+} Detection

ROS and Ca^{2+} levels were detected using DCFH-DA and Fluo-4 AM reagent (Beyotime) respectively according to the manufacturer’s instructions. After incubated for 48 hours, the supernatant was removed and cells were washed with PBS for three times. 100 μL DCFH-DA

or Fluo-4 AM working solution was added to each well and incubated at 37℃ for 20 minutes. Then the cells were washed with PBS for 3 times and images were acquired using an ImageXpress Micro® Confocal High-Content Imaging System (Molecular Devices). The mean fluorescence intensity of cells positive for DCFH-DA or Fluo-4 AM staining was determined.

4. Histology and Immunohistochemistry (IHC)

At the end of the experiment, mice were sacrificed and the hearts were harvested, fixed in 4% formalin, and paraffin embedded. Fibrosis was detected with Masson's trichrome staining kit (Sigma) according to the manufacturer's protocol. SGK1 expression of cardiomyocytes was determined by staining cells with antibodies specific for SGK1.

5. RT-PCR of mRNA levels

RT-PCR was performed to validate DEGs in the FoxO signaling pathway in response to Ext.R and also HBT's effect on hypertrophic cardiomyocytes stimulated with phenylephrine. Total RNA isolation, reverse transcription and quantitative RT-PCR were performed using an Ultrapure RNA Kit, a HiFiScript First Strand cDNA Synthesis Kit, and an UltraSYBR Mixture Kit (CWBIO) respectively, according to the manufacturer's instructions. Relative target gene mRNA expression level was normalized to GAPDH. Primer sequences of genes used for RT-PCR analysis are given in **Table S6**.

6. Pharmacokinetics studies

Twelve male Sprague-Dawley rats, weighing 250 ± 10 g, were purchased from Huafukang biological technology (Beijing, China). All rats were kept at the animal center of Tianjin University of Traditional Chinese Medicine (TUTCM) where temperature and humidity were maintained at 25 ± 5 °C and 50 ± 5%, respectively and a 12 h dark-light cycle. The animal study was strictly conducted following guidelines for the care and use of laboratory animals and the related ethical regulations of TUTCM. Rats were allowed free access to food and water and made to acclimatize for 7 days, and were abrosia for 12 hours before experiment. They were then randomly divided into two groups. All rats were orally administered 40 mg/kg herbacetin (0.5% CMCNa aqueous solution, 4 mg/mL) to determine pharmacokinetic

behavior of herbacetin and its tissue distribution separately. After treatment, about 200 μL blood samples were drawn from suborbital venous of each rat into heparinized polythene tubes at 5, 10, 15, 30, 45, 60, 120, 180 and 240 min. The blood samples were then centrifuged at 7000 rpm for 10 min. In tissue distribution group, after administration for 15 min, animals were euthanized by chloral hydrate and tissues (kidney, heart and lung) were collected.

7. Western blot.

The proteins in the lysate were separated on 10% SDS polyacrylamide gels, transferred to PVDF membranes (Millipore, Boston, MA), and stained with the related primary antibodies (1:1000). Primary antibodies were stained with HRP-conjugated secondary antibodies and visualized with a chemiluminescence ECL Western-blotting system (Bio-Rad Laboratories, USA). The values of protein density were normalized to Sham group on the same membrane in Western blot analysis.

8. Statistical Analysis

The data were interpreted as means ± SD of three or more independent experiments. Statistical analysis with unpaired two-tailed t test for comparisons between two groups and one-way analysis of variance (ANOVA) and Dunnett correction for comparisons among more than two groups were carried out using GraphPad Prism (GraphPad Software, SanDiego, CA, USA). P-values less than 0.05 were considered statistically significant.

III. Supplementary tables for methods

Table S6. Sequences of primers used for RT-PCR analysis and plasmid constructions

Gene	Forward	Reverse
SGK1(Human)	GACTGTGGACTGGTGGTG	CAGGCTCTTCGGTAAACT
TNFSF10(Human)	GAGTATGAACAGCCCCT	GTTGCTTCTTCCTCTGGT
GABARAPL1 (Human)	TCTGGACAAGAGGAAGTACCTAGTGC	AGAATAAGGCGTCCTCAGGTCTCAG
GADD45A(Human)	TCGGCTGGAGAGCAGAAGACC	ACATCTCTGTCGTCGTCCTCGTC
BCL2L11(Human)	GAGATATGGATCGCCCAAGA	GTCTTCGGCTGCTTGGTAAT
GAPDH(Human)	CAGGAGGCATTGCTGATGAT	GAAGGCTGGGGCTCATTT
ANP(Rat)	GGGAAGTCAACCCGTCTCA	GGCTCCAATCCTGTCAATCC
BNP(Rat)	TGGGCAGAAGATAGACCGGA	ACAACCTCAGCCCGTCACAG
GAPDH(Rat)	GACATGCCGCCTGGAGAAAC	AGCCCAGGATGCCCTTTAGT
Flag-SGK1(D177A)	Forward: TTTGTCCTAGCCTACATTAATGGTGGAGAGTTGTTC<br>Reverse: CATTAATGTAGGCTAGGACAAAGTACAATTTGTCAGC	
Flag-SGK1(N227A)	Forward: TAAAACCAGAGGCTATTTTGCTAGATTCACAGGGACAC<br>Reverse: CTAGCAAAATAGCCTCTGGTTTTAAGTCTCTATAAACGATG	
Flag-SGK1(T239A)	Forward: CATTGTCCTTGCTGACTTCGGACTCTGCAAG<br>Reverse: CCGAAGTCAGCAAGGACAATGTGTCCCTGTG	
